# Polymer-bridged nanofibrils in a high-molar-mass polyester *via* co-assembly of benzenetricarboxamide end groups and an additive†

**DOI:** 10.1039/d5qo00087d

**Published:** 2025-05-13

**Authors:** Sophia Thiele, Michael Giffin, Matthieu Wendling, Daniel Görl, Christopher J. G. Plummer, Holger Frauenrath

**Affiliations:** a École Polytechnique Fédérale de Lausanne (EPFL), Institute of Materials 1015 Lausanne Switzerland holger.frauenrath@epfl.ch

## Abstract

Benzenetricarboxamide (BTA) derivatives are versatile compounds widely employed as nucleating agents in commercial semicrystalline plastics and as supramolecular ligands in self-assembling telechelic polymer-based organogels, hydrogels, and bulk elastomers. However, their effectiveness as supramolecular modifiers is typically limited to low-molar-mass apolar polymers. Here, we report the supramolecular aggregation of a BTA-end-functionalized semicrystalline aliphatic polyester with a number-average molar mass several times its entanglement molar mass, blended with a matching low-molar-mass BTA additive. In these blends, the BTA end groups and additive co-assemble to form a new phase comprising a network of polymer-bridged nanofibrils. This network gives rise to a high-melt-strength rubbery regime that is absent from the pure telechelic polyester but extends to temperatures well above its melting point in the blends. Moreover, the nanofibrils prove to be highly efficient nucleating agents for crystallization of the polyester, significantly outperforming bulk additive precipitates. Our findings hence demonstrate that the co-assembly of polymer end groups with a low-molar-mass additive may facilitate supramolecular aggregate formation in polymer matrices where end-modification alone is insufficient, leading to materials with increased melt strength, crystallization rates, thermal dimensional stability, and valuable benefits for industrial applications.

## Abstract 2

Co-assembly of a BTA end-functionalized high-molar-mass polyester with a matching BTA additive yields polymer-bridged nanofibrils that result in rubber-like melts, offering promise for enhanced processing of sustainable plastics.

## Graphical abstract

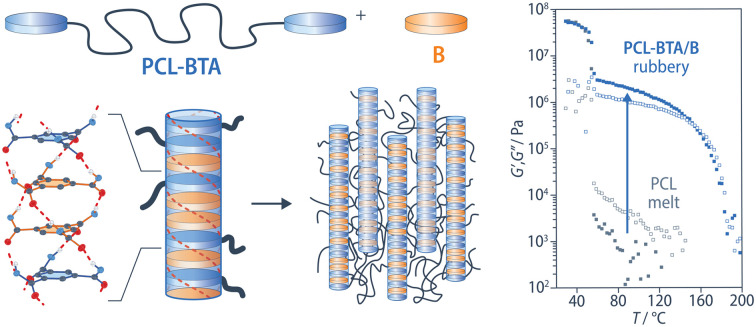

## Introduction

Supramolecular functional motifs that self-assemble in a controlled and reversible manner into complex hierarchical structures^1–3^ are used in a wide range of applications, including soft robotics,^4,5^ targeted drug delivery systems,^6^ soft pliable adhesives,^7^ and tissue-mimicking materials.^8^ An important class of materials based on such motifs are supramolecular polymers, which have evolved from small molecules that aggregate into materials with polymer-like properties,^9–13^ to telechelic polymers end-modified with supramolecular functional motifs,^14,15^ improving toughness, enabling self-healing, and facilitating recycling.^10,16–21^

Many of the supramolecular polymers and networks based on telechelic polymers reported are derived from multivalent hydrogen-bonded motifs that we refer to here as “inherently monotopic”, that is, ligands designed to form dimers with one complementary partner. Examples of such ligands include the self-complementary 2-ureido-4-pyrimidone (UPy) motif,^9^ and hetero-complementary donor/acceptor systems such as uracil/2,6-diacyl-amino-pyridine,^22,23^ other nucleobases,^24^ UPy/2,7-diamido-1,8-naphthyridine,^25,26^ Hamilton receptor/barbituric acid,^27–30^ and isophthalic acid/pyridine.^31^ In some cases, these dimers may further undergo microphase separation to form larger clusters with irregularly shaped domains analogous to those observed in thermoplastic elastomers containing hard and soft segments.^32^

By contrast, “inherently ditopic” hydrogen-bonded motifs bind simultaneously to two other ligands to form one-dimensionally extended aggregates with well-defined lateral dimensions on the nanometer scale.^33,34^ The vast majority of such ditopic motifs rely on multivalent hydrogen bonding between amide,^35–39^ urethane,^7,40^ or urea^41–43^ functionalities that show cooperative self-assembly due to electronic coupling in the extended N–H⋯O=C hydrogen bond strands within the aggregates.^2,12,44^ In polymers end-modified with UPy ligands with additional lateral ditopic urea^45^ or urethane^46^ motifs, for instance, it is the combination of the ditopic hydrogen bonds of the urea or urethane motifs and the π-interactions between UPy units that promote the formation of one-dimensionally extended aggregates with well-defined lateral dimensions.^47^ Telechelic polymers modified in this way are reported to show significantly increased tensile strength.^45,46^ These examples highlight the importance of synergistic and/or competing interactions for the design of materials with precisely controlled, hierarchical morphologies.^48^

Well-established supramolecular motifs for the reliable formation of nanofibrils include β-sheet-forming oligopeptides,^35,49,50^ oligourethanes,^51^ oligoureas,^52^ and their hybrids,^40,53–55^ as well as benzene-1,3,5-tricarboxamide (BTA) derivatives.^37,38^ The last of these have been shown to form nanofibrils by three-fold hydrogen bonding of the amide functions combined with π-interactions between the benzene cores, resulting in extended 1D stacks. The geometric discrepancy between the length of the constituting unit involved in C–N–H⋯O=C hydrogen bonding and the π-stacking distance causes a rotational offset of BTA units along the stack, leading to a helical arrangement of the peripheral amide functions.^38,56^ This disrupts lateral aggregation and crystallization into hexagonal or pseudo-hexagonal columnar phases in dilute solutions of BTA derivatives^57^ and BTA-functionalized polymers^37,58^ in favor of nanoscopic BTA aggregates. For instance, apolar low-molar-mass polymers, such as poly(ethylene-*ran*-butylene), which otherwise behave as viscous fluids, become soft elastomers when end-modified with BTA motifs.^37^ By contrast, BTA end-group aggregation is not observed in polymers with higher polarity, such as aliphatic polyesters.^59^

The self-assembly of end groups also generally becomes less favorable with increasing polymer molar mass.^42^ Previous investigations of supramolecular polymers and networks have therefore largely excluded polymers with molar masses high enough for the formation of a robust entanglement network. This is a critical limitation given that entanglement is generally key to high extensibility and toughness in the solid state,^60,61^ and the melt properties required for industrially relevant processing techniques, such as thermoforming, film blowing, and extrusion.^60,62–64^ The few notable exceptions make use of specific polymer architectures to increase the local concentration of aggregating units. For instance, materials with increased toughness are obtained from high molar mass polyisoprene (PI) block copolymers in which the central PI block is well above its entanglement molar mass and the terminal blocks are modified with oligoalanine side groups so that their high local concentration renders aggregation favorable.^65,66^ By contrast, when the aggregating motifs are incorporated into the corresponding random copolymers, the reduced segment length between physical crosslinks results in materials with reduced tensile strength and extensibility.^65^

Unlike supramolecular networks based on telechelic polymers end-modified with inherently monotopic ligands, which tend to break down upon the addition of a low-molar-mass ligand,^9^ the high-aspect-ratio, one-dimensionally extended aggregates formed by intrinsically ditopic end groups may be significantly reinforced by co-assembly with a corresponding low-molar-mass ligand. For instance, co-assembly of a low-molar-mass BTA derivative with BTA-modified polyethylene glycol (PEG) telechelics, which form a micellar fluid in the absence of the additive, has been reported to result in stable viscoelastic hydrogels containing fibrillar domains.^67,68^

We recently demonstrated that a similar approach was effective for high-molar-mass polymers where the end group concentration might otherwise be insufficient for aggregation to occur. To this end, we used the co-assembly of polymer end groups and a low-molar-mass additive, both based on a β-sheet-forming oligopeptide motif, to create a robust supramolecular network in poly(ε-caprolactone) (PCL), with a molar mass several times the entanglement threshold.^50^ The resulting high melt elasticity, melt extensibility, and melt strain hardening enable the preparation of highly oriented films by melt drawing, with a correspondingly high room-temperature yield stress.^50^ However, to the best of our knowledge, the analogous use of a low-molar-mass BTA additive to modify the viscoelastic properties of well-entangled BTA-end-functionalized polymers in the bulk melt state has not yet been reported.

Low-molar-mass BTA derivatives are used as ferroelectric switches,^69^ emitter materials in organic diodes,^70^ organogelators,^71,72^ and hydrogel-promoters with low cytotoxicity for tissue engineering.^58,68,73,74^ They are of particular interest in the plastics industry as nucleating agents for the crystallization of semicrystalline polymers such as isotactic polypropylene (iPP),^75–77^ polyvinylidene fluoride (PVDF),^78^ polybutylene terephthalate (PBT),^79^ polylactic acid (PLA),^80–82^ and the semiconductor poly(3-hexylthiophene) (P3HT).^83^ BTA nucleating agents are hence important processing additives in many packaging materials, and some are considered safe for use in contact with food by the US Food and Drug Administration (FDA) and the European Food Safety Authority (EFSA).^84^ They typically show high-aspect-ratio morphologies, for instance, needle-like crystals, and their nucleation efficiency increases with their specific active surface area.^85^ Systematic studies of the concentration dependence of BTA structure formation and its influence on polymer crystallization have been used to construct phase diagrams for binary polymer/BTA nucleating agent blends.^86^ However, blends of BTA derivatives with telechelic polymers modified with end groups that potentially co-assemble to form nanoscopic structures with significantly higher surface-to-volume ratios than conventional BTA nucleating agents, have not yet been investigated for their nucleation efficiency.

**Fig. 1:**
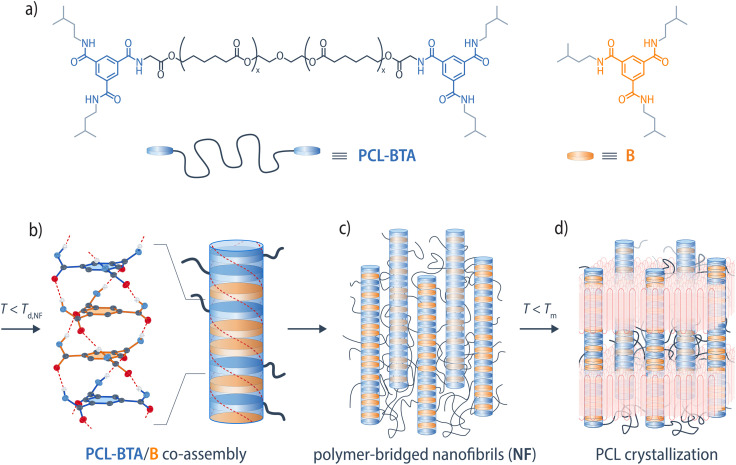
(a) Chemical structure of end-modified PCL-BTA, and matching additive B. Upon cooling, the end groups of PCL-BTA/B co-assemble with B by three-fold hydrogen bonding into (b) polymer-tethered nanofibrils comprising one-dimensional stacks of BTA end groups and additive molecules. (c) In the bulk materials, the nanofibrils are bridged by the polymer segments and (d) coexist with crystalline PCL lamella upon cooling to *T* < *T*_m_. For clarity, the alkyl side chains of B have been omitted in (b–d).

In the present work, we explore the behavior of PCL-BTA, a high-molar-mass PCL end-modified with a BTA-based motif, in the presence of the matching low-molar-mass BTA derivative B (Fig. 1). We establish a phase diagram for this binary system and show that, below a certain threshold B concentration, co-assembly of the PCL-BTA end groups with B results in the precipitation of nanophase-separated fibrillar BTA aggregates upon cooling from the melt. In this low-concentration regime, the nanoscopic fibrillar BTA aggregates act as more efficient nucleating agents for PCL crystallization than the bulk BTA precipitates observed in reference blends of B with unmodified PCL. Moreover, the physical network formed by the nanofibrils and PCL manifests itself as a rubbery plateau in dynamic shear rheometry temperature sweeps that extends to well above the PCL crystallization temperature. This leads to improved thermal dimensional stability and increased melt elasticity, which facilitates thermoforming. We hence overcome previous limitations for the assembly of BTA-based end groups in high-molar-mass, polar polymers.

## Results and discussion

We have carried out a systematic investigation of blends of the low-molar-mass additive *N*,*N*′,*N*′′-tris(isopentyl)-1,3,5-benzenetricarboxamide (B) with PCL-BTA, a telechelic PCL end-modified with *N*′,*N*′′-bis(isopentyl)-1,3,5-benzenetricarboxamide ligands, the overall number-average molar mass of which, *M̄*_n_ = 110 000 g mol^−1^, is about 37 times the entanglement molar mass of PCL (*M*_e_ ≈ 3000 g mol^−1^).^87^ We focus on the phase behavior, melt properties, thermal dimensional stability, and nucleation efficiency of these materials, using blends of B with unmodified PCL of the same molar mass as reference materials.

PCL is an aliphatic, semi-crystalline polyester with a degree of crystallinity of about 40%, a glass transition temperature, *T*_g_, of −62 °C (measured by DSC at a cooling rate of 10 °C min^−1^), and a nominal melting temperature, *T*_m_, of about 58 °C, above which it transforms into a low-viscosity liquid for the range of molar masses considered here (ESI Fig. S1†). The low melt strength of PCL is incompatible with many important industrial processes, and its inadequate dimensional stability at temperatures above *T*_m_ has largely restricted its use to biomedical applications.^88^

We begin by discussing the behavior of the PCL/B reference blends in order to establish a benchmark for the interpretation of the results for the modified blends. The additive B used in our study forms extended 1D stacks in its bulk state at room temperature, in common with a wide range of other BTA derivatives. These stacks adopt a pseudo-hexagonal parallel close-packed crystalline structure with a monoclinic unit cell, the lattice parameters of which vary somewhat depending on the crystallization conditions, characterized by strong Bragg peaks with *d*-spacings, *d*_B_, of around 1.3 nm and intercolumnar spacings in the range of 1.25–1.55 nm. Upon heating, this structure first undergoes an endothermal transition to a columnar mesophase at *T*_meso_ = 207 °C, followed by a transition to the isotropic melt at its dissociation temperature, *T*_d0_ = 269 °C (ESI Fig. S2†), consistent with previous observations.^89^

**Fig. 2:**
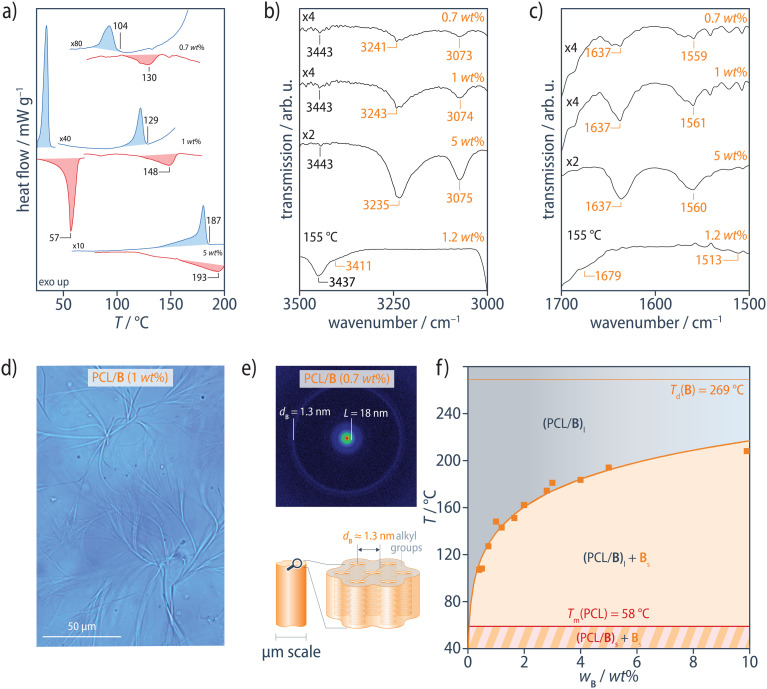
(a) DSC heating (red) and cooling (blue) scans of PCL/B at 10 °C min^−1^ for different B concentrations. For clarity, the PCL melting and crystallization transitions at *T*_m_ = 58 °C and *T*_c_ = 35 °C, respectively, are only shown for 1 wt% B. (b) ATR FTIR spectra at room temperature show the N–H stretching band at 3235–3243 cm^−1^, (c) the C=O stretching band at 1637 cm^−1^, and the combined N–H bending/C–N stretching band at 1559–1561 cm^−1^, characteristic of strongly hydrogen-bonded amides in extended aggregates of B, all of which shift to positions corresponding to free amides^90^ in FTIR transmission spectra recorded at 155 °C from a film of PCL/B (1.2 wt%) on KBr. (d) Optical micrographs show micrometer-sized fibrous precipitates of B, formed by lateral aggregation of the individual stacks, giving (e) Bragg reflections in the neighborhood of *q* ≈ 0.24 Å^−1^ (*d*_B_ ≈ 1.3 nm) in XRD patterns obtained at room temperature. (f) Phase diagram for the PCL/B reference blends, constructed from *T*_d_ determined from DSC heating scans. The PCL/B blends form an optically transparent, homogeneous melt, (PCL/B)_l_, at sufficiently high temperatures. Solid bulk additive domains (microfibers), B_s_, precipitate from the liquid phase upon cooling and persist to below the polymer melting temperature (58 °C), where crystalline lamellae begin to form in the PCL matrix (PCL/B)_s_. The solid orange curve represents fitting of the Flory–Huggins model to the data. See ESI Figs. S3–S7† for more information.

In the range of 0.5–10 wt%, the PCL/B reference blends show an intense exothermic transition in DSC cooling scans at 10 °C min^−1^, corresponding to crystallization of PCL at *T*_c_ = 35 °C, but also an additional weaker exothermic transition at higher temperatures, *T*_agg_, corresponding to the aggregation of B (Fig. 2a and ESI Fig. S3†). Subsequent heating scans reveal the PCL melting transition at *T*_m_ = 58 °C and an endothermic transition at higher temperatures, *T*_d_, attributed to dissociation of the B precipitates. Dissociation nevertheless occurs at significantly lower temperatures than that for the pure additive B (*T*_d0_ = 269 °C) and blends of B with isotactic polypropylene for a given B concentration,^86^ owing to the comparatively high solubility of B in the polar PCL matrix. *T*_agg_ and *T*_d_ both increase systematically with B concentration, but *T*_d_ is 6–26 °C higher than *T*_agg_, with the highest supercoolings being observed at the lowest B concentrations. Aggregation of B is therefore inferred to be kinetically controlled under these conditions, so that *T*_d_ is assumed to be more representative of equilibrium conditions than *T*_agg_.

In Fourier transform infrared (FTIR) spectra recorded in the attenuated total reflection (ATR) configuration at room temperature, the N–H stretching band at 3232–3240 cm^−1^, the C=O stretching band at 1637–1641 cm^−1^, and the combined N–H bending/C–N stretching band at 1560–1561 cm^−1^ are characteristic of strongly hydrogen-bonded BTA aggregates in the PCL/B blends, regardless of the B concentration (Fig. 2b, c and ESI Fig. S4†).^89,90^ FTIR spectra recorded in transmission mode at temperatures above *T*_d_ show the N–H stretching band at 3411 cm^−1^, the C=O stretching band at 1679 cm^−1^, and the combined N–H bending/C–N stretching band at 1513 cm^−1^, all of which indicate the presence of free amide groups, as described previously for BTA derivatives in dilute solution (ESI Fig. S5†).^89^B may hence be assumed to be homogeneously dispersed in the PCL matrix above *T*_d_.

Consistent with the DSC results, solid bulk precipitates, B_s_, in the form of microfibers are observed by bright-field optical microscopy (OM) between *T*_agg_ and *T*_c_ during cooling (Fig. 2d and ESI Fig. S6†), but are obscured by the PCL crystallinity at temperatures below *T*_c_, (PCL/B)_s_, and disappear upon heating to above *T*_d_, where OM indicates a single-phase, amorphous melt, (PCL/B)_l_, for the range of B concentrations investigated. Room-temperature X-ray diffraction (XRD) patterns of all the blends show an intense Bragg reflection at *q* < 0.2 Å^−1^, which corresponds to the lamellar long period of PCL, *L*, of 17.5–18.3 nm.^91^ The additional, markedly less intense Bragg reflections in the neighborhood of *q* ≈ 0.24 Å^−1^ (*d*_B_ ≈ 1.3 nm) are characteristic of the bulk B crystal structure (Fig. 2e and ESI Fig. S7†).^89^

The *T*_d_ values determined from DSC heating scans were fitted using the Flory–Huggins model, as described in our previous study of oligoalanine aggregates in PCL,^50^ assuming a Flory–Huggins interaction parameter in the form of1
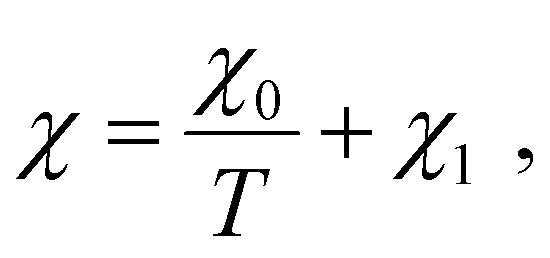
where *χ*_0_ = 1633 K and *χ*_1_ = –2.569 are fitting parameters and the reference volume is that of B (Fig. 2f). When normalized with respect to the corresponding molar masses, *χ* for B is found to be a factor of 1.5 lower at 70 °C than *χ* determined in the same way for the oligoalanine aggregates in PCL,^50^ indicating B to be significantly more soluble in PCL than the oligoalanines.

The end-modified PCL-BTA used in the investigation of the corresponding supramolecular PCL-BTA/B blends is obtained from commercial telechelic PCL with a degree of (difunctional) hydroxyl end-group functionalization of at least 92%, determined by comparing *M̄*_n_ obtained from gel permeation chromatography (GPC) with the end-group content according to the peak integrals in ^1^H nuclear magnetic resonance (NMR) spectroscopy (ESI Fig. S8†). The hydroxyl end groups were then quantitatively modified by Steglich esterification with Fmoc-protected glycine, as confirmed by the absence of the peak at *δ* = 3.63 ppm assigned to the C**H**_**2**_–OH end groups and the presence of peaks characteristic of the Fmoc protection group in the range of *δ* = 7–8 ppm in the ^1^H NMR spectrum of PCL-GlyFmoc (ESI Fig. S9†). Subsequent deprotection yields NH_2_-terminated PCL, which is functionalized *via* a standard peptide coupling reaction with 3,5-bis(isopentylcarbamoyl)benzoic acid (ESI Scheme S1†). By comparing *M̄*_n_ obtained from GPC with the end-group content determined from peak integrals in ^1^H NMR spectroscopy, we estimate a degree of BTA end-group functionalization of at least 95% (ESI Fig. S10†). Pure PCL-BTA, which has an end-group concentration of 0.63 wt%, shows no evidence of end-group aggregation and its melt behavior is therefore very similar to that of unmodified PCL of the same molar mass (ESI Fig. S1c and d†), as generally observed for high-molar-mass polymers^42,50^ and low-molar-mass polymers with high backbone polarity, including polyesters.^59^

**Fig. 3:**
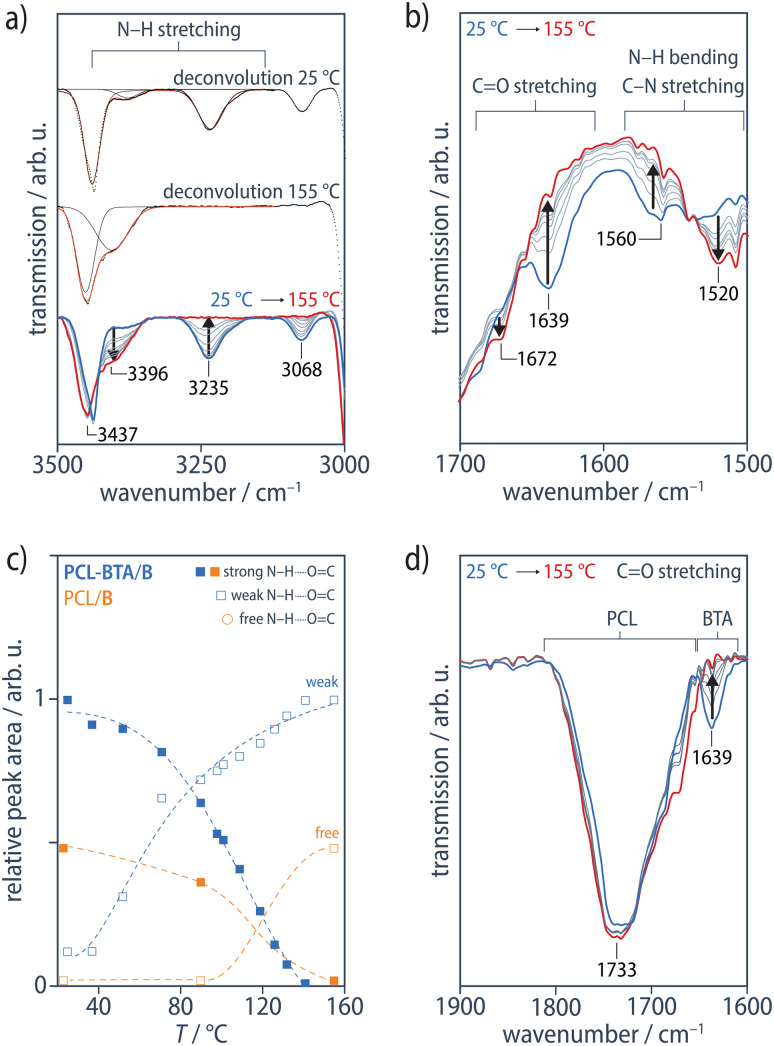
FTIR transmission spectra of a PCL-BTA/B (1 wt%) film on KBr, recorded at 25, 79, 101, 119, 126, 132, and 155 °C, showing (a) the N–H stretching band, with examples of the deconvolution of the spectra at 25 and 155 °C, and (b) the C=O stretching band and the combined N–H bending/C–N stretching band. The ensemble of deconvoluted spectra is shown in ESI Fig. S11.† The sharp absorption signal visible in all the spectra at 3435–3445 cm^−1^ is assigned to water absorbed by PCL.^92^ (c) The area of the N–H stretching peak obtained from the deconvoluted spectra of PCL-BTA/B (1 wt%) (blue) and PCL/B (1.2 wt%) (orange), normalized with respect to the area of the PCL C–H_2_ stretching peak (not shown). The dashed curves serve as guides to the eye. (d) The C=O stretching band of the PCL backbone does not change with temperature.

In what follows, unless mentioned otherwise, we compare PCL/B and PCL-BTA/B blends with equal *w*_B_, given in wt% (for the corresponding effective total concentration of BTA-based moieties, *w*_B__+__BTA_, in wt%, [B] and [B + BTA] in mol L^−1^, and the additive-to-end-group ratio, [B]/[BTA], in mol mol^−1^, see Table 1 in the Materials and methods). Upon addition of B at concentrations in the range of 0.6–5 wt% investigated, the resulting PCL-BTA/B blends again form a single-phase, amorphous melt (PCL-BTA/B)_l_ at sufficiently high temperatures, based on their homogeneous appearance in OM. However, FTIR spectra recorded of PCL-BTA/B (1 wt%) in the melt state at temperatures up to 155 °C (Fig. 3) now show the main N–H stretching band at 3396 cm^−1^, the C=O stretching band at 1672–1677 cm^−1^, and the combined N–H bending/C–N stretching band at 1520–1524 cm^−1^, indicating weakly hydrogen-bonded BTA amides, as reported previously for optically homogeneous melts of pure BTA-derivatives.^89^ The C=O stretching band assigned to the polymer backbone ester groups at 1733 cm^−1^ remains unchanged over the whole temperature range, showing that these ester groups do not interact significantly with B (Fig. 3d). Hence, in marked contrast with the PCL/B reference blends, the IR results for the modified PCL-BTA/B blends suggest that at temperatures immediately above the *T*_d_ implied by DSC scans, B is present in the form of colloidal aggregates, characterized by a weak, discontinuous hydrogen bond network, and stabilized by the end-modified PCL-BTA, which acts as a surfactant. Hence, under these conditions, *T*_d_ does not mark a transition to a fully dispersed state but rather a melting transition to a disordered, but still phase-separated state.

**Table 1:** [B] in mol L^−1^ (assuming *ρ*_PCL_ = 1140 g L^−1^),^97^ the combined *w*_B__+__BTA_ for the additive and end groups in wt% (assuming *M* = 346.15 g mol^−1^ for the BTA end group), [B + BTA] in mol L^−1^, and the resulting additive-to-end-group ratio, [B]/[BTA], in mol mol^−1^ for selected blends with various B contents in wt%

*w*_B_ wt%	PCL/B	PCL-BTA/B		
	[B] (mol L^−1^)	*w*_B__+__BTA_ (wt%)	[B + BTA] (mol L^−1^)	[B]/[BTA] (mol mol^−1^)
0	0	0.63	0.02	0
0.6	0.02	1.23	0.04	0.79
1	0.03	1.63	0.05	1.32
2	0.05	2.63	0.08	2.63
4	0.11	4.63	0.13	5.27

**Fig. 4:**
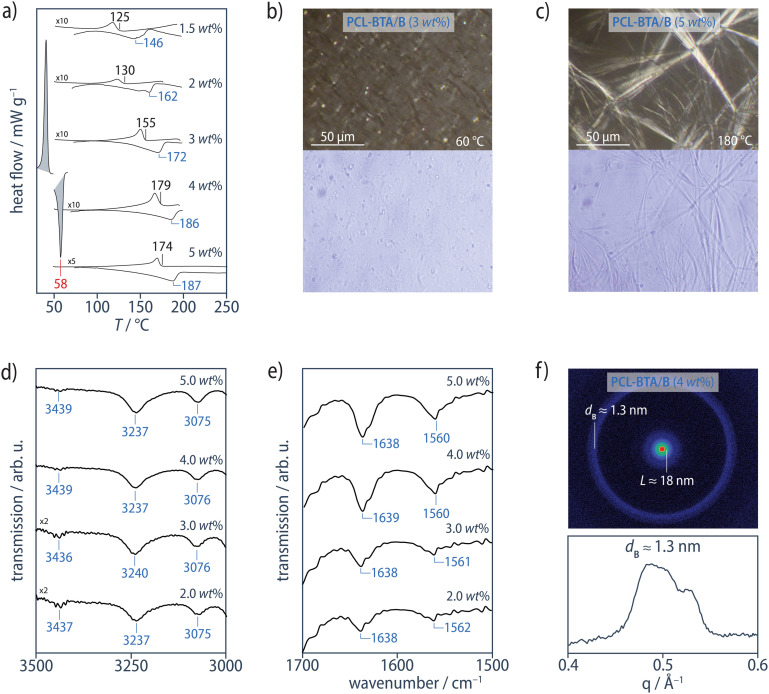
(a) DSC heating and cooling scans (10 °C min^−1^, exothermal transitions up) from the PCL-BTA/B blends for B concentrations ≥ 1.5 wt%. For clarity, the PCL melting and crystallization transitions at *T*_m_ = 58 °C and *T*_c_ = 35 °C, respectively, are only shown for one composition. (b and c) Cross-polarized (top) and bright-field (bottom) optical micrographs of PCL-BTA/B recorded at *T* < *T*_agg_ during cooling at 10 °C min^−1^ from the melt state. PCL-BTA/B blends containing ≥1.5 wt% B show microfibrous precipitates (see also ESI Fig. S12†). (d) The N–H stretching band, and (e) the C=O stretching band and the combined N–H bending/C–N stretching band from ATR FTIR spectra of PCL-BTA/B recorded at 25 °C. The sharp absorption signal visible in all the spectra at 3435–3445 cm^−1^ is assigned to water absorbed by PCL.^92^ (f) The corresponding structures show Bragg reflections in the neighborhood of *q* ≈ 0.24 Å^−1^ (*d*_B_ ≈ 1.3 nm) in XRD patterns obtained at room temperature.

DSC scans of the PCL-BTA/B blends reveal two distinct types of behavior depending on the B concentration. In a high-concentration regime corresponding to *w*_B_ ≥ 1.5 wt% ([B]/[BTA] ≥ 1.98 mol mol^−1^, see Materials and methods), we observe results similar to those of the PCL/B reference blends, that is, an exothermic transition at *T*_agg_ > *T*_c_ in cooling scans and an endothermic transition at *T*_d_ > *T*_m_ in subsequent heating scans, corresponding to association and dissociation of B aggregates, respectively (Fig. 4a). OM shows that this leads to solid bulk additive precipitates, B_s_, in the form of microfibers similar to those observed in the PCL/B reference blends according to XRD and FTIR (Fig. 4b–f, ESI Fig. S12†). The absolute values of *T*_d_ of the PCL-BTA/B blends are also similar to those of the reference blends, indicating the end groups to have little effect on the global solubility of B in PCL-BTA. *T*_agg_ values determined from the DSC cooling scans at a cooling rate of 10 °C min^−1^ are some 13–21 °C lower than the corresponding *T*_d_ in this concentration regime; the highest supercoolings are observed at B concentrations immediately above 1.5 wt%.

**Fig. 5:**
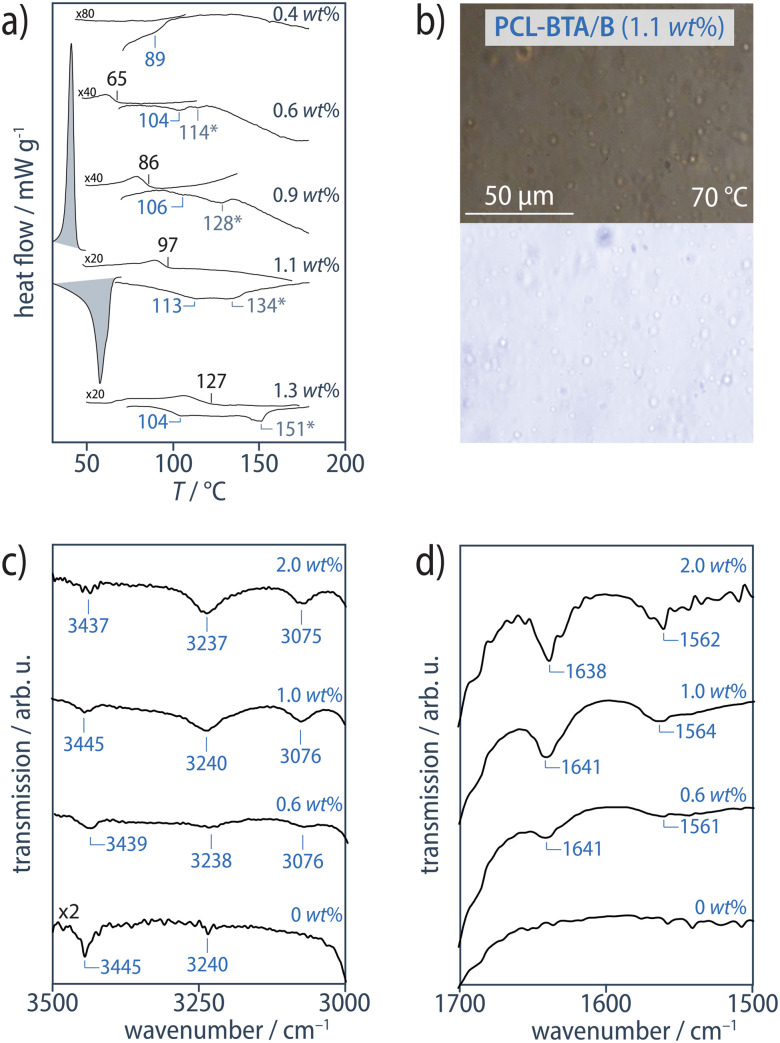
(a) DSC heating and cooling scans (10 °C min^−1^, exothermal transitions up) from PCL-BTA/B blends. For clarity, the PCL melting and crystallization transitions at *T*_m_ = 58 °C and *T*_c_ = 35 °C, respectively, are only shown for one composition. (b) Cross-polarized (top) and bright-field (bottom) optical micrographs of PCL-BTA/B recorded at *T* < *T*_agg_ during cooling at 10 °C min^−1^ from the melt state. PCL-BTA/B blends at *w*_B_ ≤ 1.1 wt% remain optically homogeneous (see also ESI Fig. S12†), but ATR FTIR spectra of PCL-BTA/B recorded at 25 °C contain (c) the N–H stretching band and (d) the C=O stretching band and the combined N–H bending/C–N stretching band indicative of BTA aggregates at all B concentrations investigated. The sharp absorption signal visible in all the spectra at 3435–3445 cm^−1^ is assigned to water absorbed by PCL.^92^

Despite these similarities in the high-concentration regime, the modified PCL-BTA/B blends behave very differently from the unmodified PCL/B reference blends in the low-concentration regime corresponding to *w*_B_ ≤ 1.1 wt% ([B]/[BTA] ≥ 1.45 mol mol^−1^, see Materials and methods), where two endothermic transitions are observed in the DSC heating scans at temperatures above *T*_m_. The first of these transitions occurs at temperatures 18–35 °C below *T*_d_ in PCL/B for a given B concentration (Fig. 5a), which we refer to as *T*_d,NF_, and is identified by the co-aggregation of B with the chain ends to form a distinct nanofibrillar phase upon cooling. Although OM suggests the melt remains optically homogeneous at temperatures down to *T*_c_ for PCL upon cooling and immediately above *T*_m_ upon heating (Fig. 5b, ESI Fig. S12†), FTIR spectra exclusively show sharp absorption signals at about 3238 cm^−1^ (N–H stretching) and 1642 cm^−1^ (C=O stretching); this proves that B and the PCL-BTA end groups nevertheless quantitatively assemble into strongly hydrogen-bonded BTA aggregates, with no indication of weakly hydrogen-bonded, disordered aggregates (Fig. 5c and d).^90^ Moreover, the ratio of the intensity of the 3235 cm^−1^ peak in the temperature-dependent IR spectra (Fig. 3c) of PCL-BTA/B (1 wt%) below 90 °C to that in PCL/B (1.2 wt%) is close to the ratio of the combined concentrations of the PCL-BTA end groups and additive B in the modified blend to the B concentration in the reference blend. This indicates that practically all the end groups in PCL-BTA/B take part in aggregation.

**Fig. 6:**
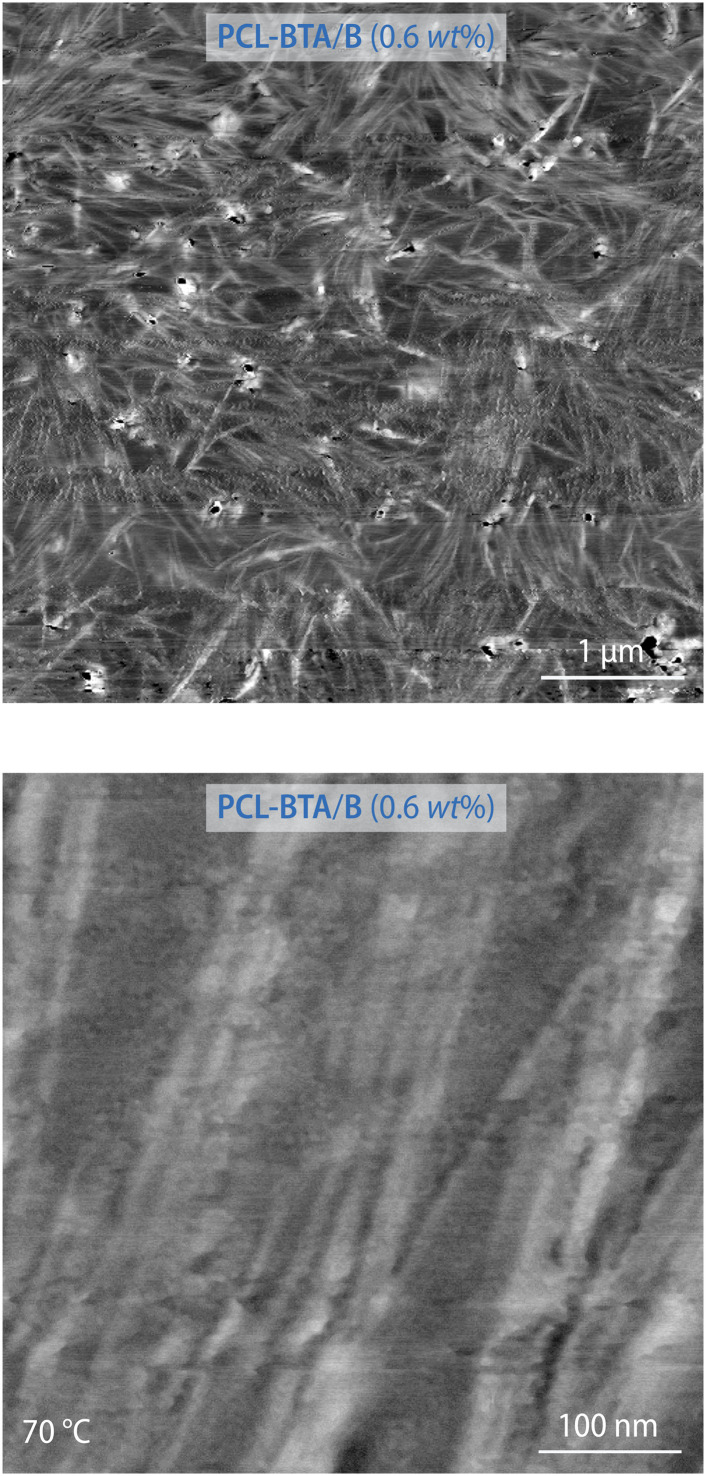
Intermittent-contact AFM phase images of PCL-BTA/B (0.6 wt%) recorded at 70 °C at different magnifications. See ESI Fig. S13.†

The nanofibrillar nature of the resulting structures is confirmed by AFM images recorded for PCL-BTA/B (0.6 wt%) heated to between *T*_m_ and *T*_d,NF_, which are dominated by high-aspect-ratio nanofibrils with diameters in the nanometer size range, bearing in mind that the radius of the curvature of the AFM tip may be up to 7 nm, locally aligned with interfibrillar spacings in the range of 10–20 nm (Fig. 6 and ESI Fig. S13†). The nanofibril spacing is hence considerably greater than the intercolumnar spacings characteristic of the crystalline bulk B precipitates, but comparable with a value of 13 nm estimated from volumetric considerations for an ideal dispersion of single stacks of co-assembled B and PCL-BTA end groups (see Materials and methods for details). However, it generally remains smaller than the root-mean-square end-to-end distance of the present PCL of about 25 nm,^93^ consistent with substantial incorporation of the chain ends in the nanofibrils and implying significant bridging of these by the PCL chains or *via* trapped entanglements.

**Fig. 7:**
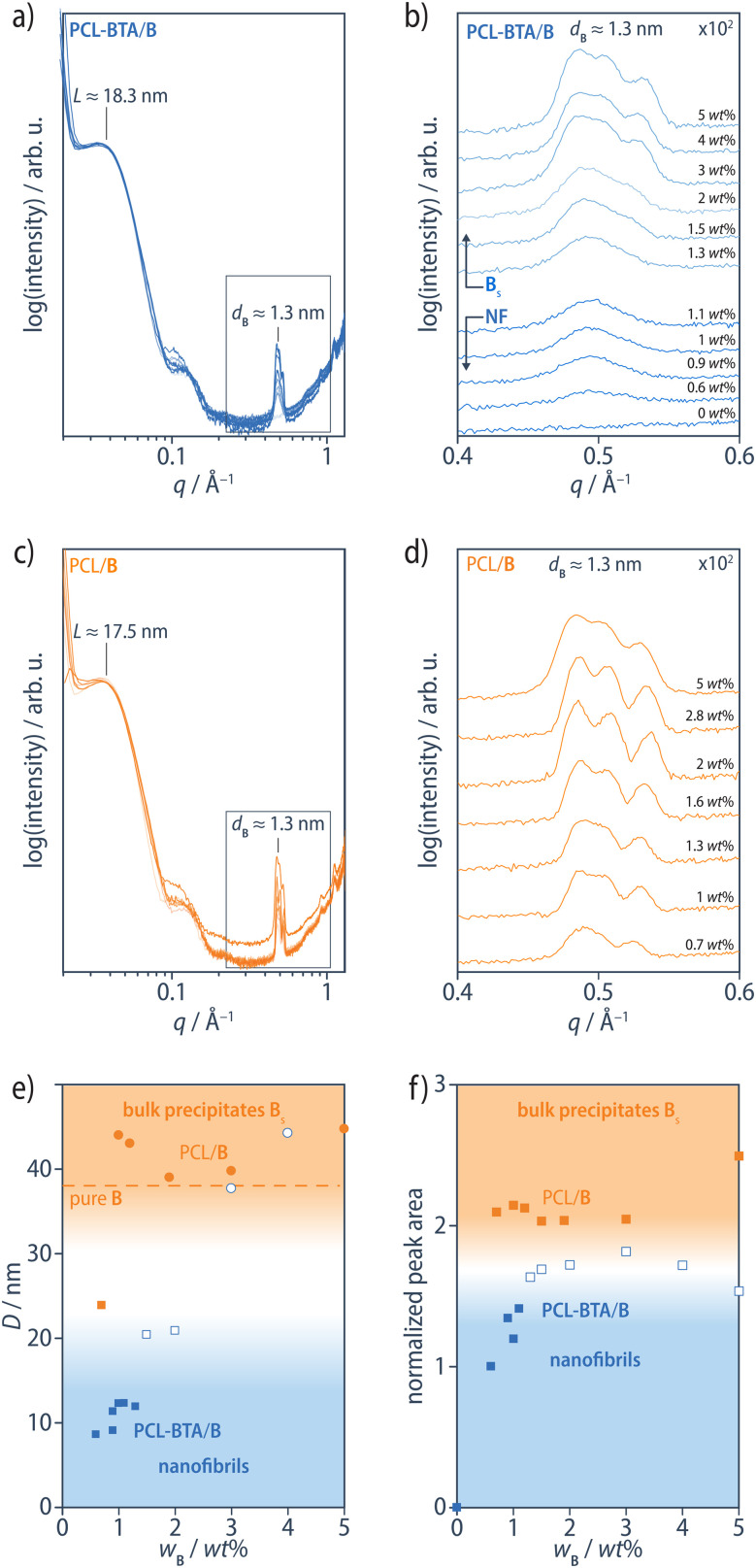
(a) 1D SAXS diffractograms of PCL-BTA/B blends, measured on flat specimens obtained by cooling from the melt at −10 °C min^−1^, normalized with respect to the intense low *q* Bragg peaks corresponding to the lamellar long period, *L*, of the PCL matrix at about 17–18 nm. (b) Enlargement of the Bragg reflections in the neighborhood of *q* ≈ 0.24 Å^−1^ (*d*_B_ ≈ 1.3 nm). (c and d) Corresponding 1D SAXS diffractograms of PCL/B blends. (e) Total area of the Bragg peaks in the neighborhood of *q* ≈ 0.24 Å^−1^ divided by the B concentration and plotted against the B concentration, and (f) the crystallite size, *D*, estimated from *β* for the Bragg reflections at *d*_B_ ≈ 1.3 nm using the Scherrer equation, eqn (2), in both the PCL/B (orange symbols) and PCL-BTA/B blends (blue symbols – filled symbols for the NF regime, open symbols for the regime in which bulk precipitates become visible in OM); the dashed line corresponds to *D* estimated from *β* for bulk B, for which extrinsic factors such as instrumental broadening are assumed be significant.

In XRD scans of PCL-BTA/B for B concentrations in the range of 0.6–1.1 wt%, Bragg peaks corresponding to bulk B precipitates are still present but overlap to form a single broad peak, in marked contrast to the PCL/B reference materials where the three reflections remain well-resolved even at the lowest B concentrations (Fig. 7a–d). The resulting relatively large effective XRD peak widths at half-maximum, *β*, may reflect reduced crystal sizes, *D*, according to the Scherrer equation2
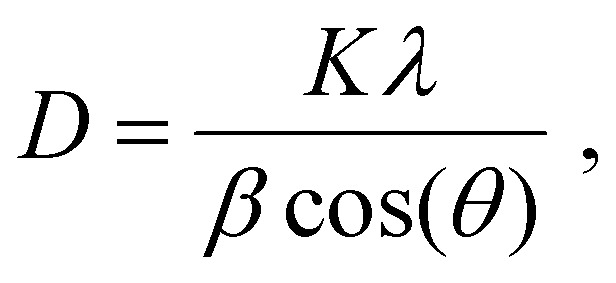
where *K* is the Scherrer constant (taken to be 0.9), *λ* = 1.54 Å is the X-ray wavelength, and *θ* is the Bragg angle. The corresponding *D* values of 9–12 nm are certainly considerably smaller than the values of 24 nm or more implied by the peak widths for PCL/B and PCL-BTA/B (≥1.5 wt%). Indeed, the largest *D* values obtained in this way are likely to be underestimates due, for instance, to instrumental broadening, as reflected by the value of only about 38 nm estimated from the Bragg peaks observed in bulk B (Fig. 7e). Moreover, the Bragg peak intensities are also significantly lower for PCL-BTA/B than for PCL/B at any given B concentration but particularly at *w*_B_ ≤ 1.1 wt% (Fig. 7f). Thus, only around half the B molecules in PCL-BTA/B (0.6 wt%) diffract at around *q* = 0.24 Å^−1^ compared with PCL/B with a similar B concentration. Given that the FTIR spectra imply nearly all the ligands form hydrogen-bonded BTA aggregates, and taking into account the end-group concentration of about 0.6 wt%, it is implied that only about 25% of the BTA stacks show significant lateral aggregation in PCL-BTA/B (0.6 wt%). This is consistent with the presence of tethered PCL chains in stacks containing the end groups, and one concludes that many of the co-assembled nanofibrils present in PCL-BTA/B at low B concentrations may comprise a single BTA stack.

**Fig. 8:**
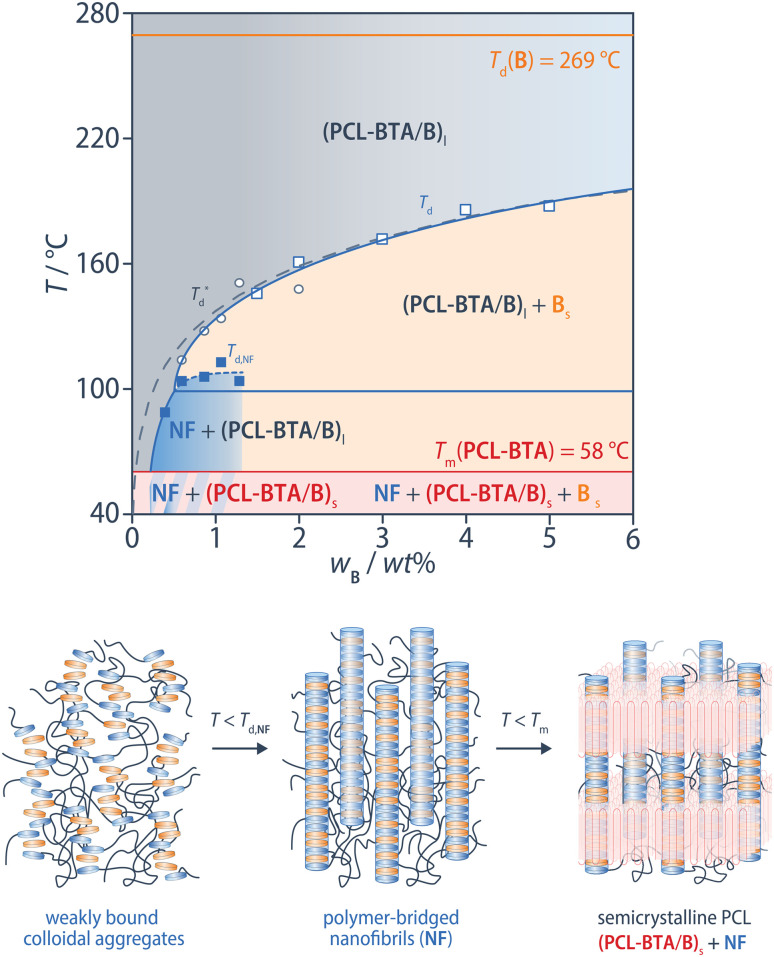
Phase diagram of PCL-BTA/B blends constructed from *T*_d_^*^ (open circles), *T*_d,NF_ (filled squares) and *T*_d_ (open squares) obtained from DSC heating scans of PCL-BTA/B blends. (PCL-BTA/B)_l_ – homogeneous melt; NF – nanofibrils; B_s_ – bulk B precipitates; (PCL-BTA/B)_s_ – solid solution. The grey dashed line indicates fitting of the Flory–Huggins model to *T*_d_ for PCL/B (see Fig. 2).

A new phase region, denoted NF and bounded by *T*_d,NF_ therefore appears in the pseudo-phase diagram for PCL-BTA/B in the B concentration range of 0.6–1.1 wt%, which gradually gives way to bulk B precipitation (bounded by *T*_d_) as the B concentration increases beyond this range. In this region, we propose that the polymer-stabilized, weakly hydrogen-bonded colloidal aggregates revealed by FTIR are converted into a phase characterized by a network of strongly hydrogen-bonded polymer-bridged nanofibrils that persist upon PCL crystallization (Fig. 8).

In addition to the dissociation transition of the nanofibrils at *T*_d,NF_, we observe an endothermic transition at higher temperatures, *T*_d_*, which is very close to *T*_d_ of the corresponding PCL/B blends for a given B concentration. However, no corresponding exothermic transition is observed in the cooling scans. In view of the supercooling associated with bulk B precipitation in the high-concentration regime, we therefore assume it to be kinetically suppressed at low B concentrations in favor of a direct transition from the PCL-BTA-stabilized colloidal aggregates to co-assembled nanofibrils. However, B molecules released from the nanofibrils upon heating through *T*_d,NF_ are inferred to reorganize into bulk precipitates that subsequently melt at *T*_d_*. It is noteworthy that the *T*_agg_ values determined from DSC cooling scans at 10 °C min^−1^ are also some 16–40 °C lower than *T*_d,NF_ in this regime (Fig. 5a). At lower cooling rates, however, *T*_agg_ approaches *T*_d,NF_, confirming that this is a kinetic effect, and the cooling rate has little effect on the values of *T*_d,NF_ and *T*_d_* observed in subsequent heating scans at 10 °C min^−1^ (ESI Fig. S14†).

**Fig. 9:**
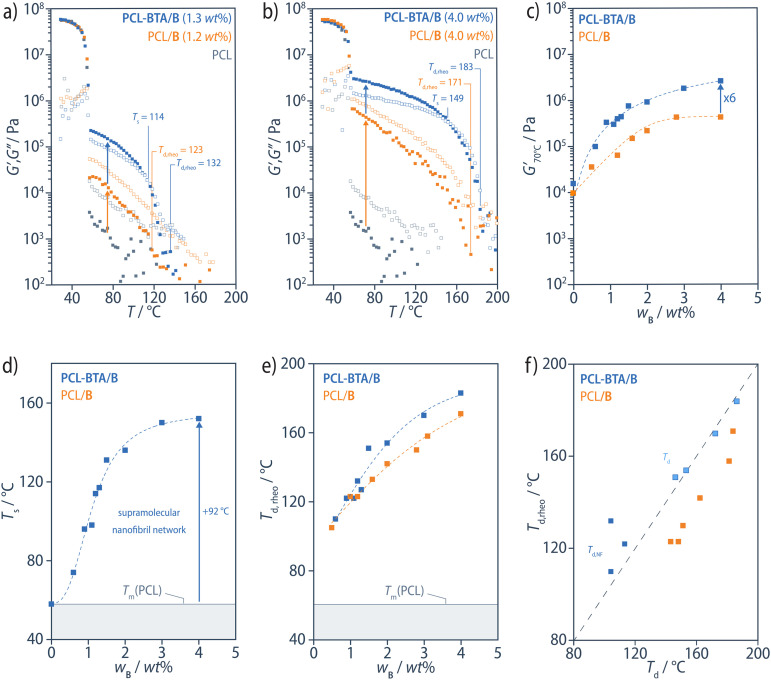
(a and b) Representative plots of the storage (*G*′, filled squares) and loss (*G*′′, open squares) moduli obtained from oscillatory shear rheometry heating scans for the PCL-BTA/B (blue) and PCL/B blends (orange) at a frequency of 1 rad s^−1^ and a scanning rate of 10 °C min^−1^. Data for other B contents are shown in ESI Fig. S15.† (c) *G*′ at 70 °C increases systematically with *w*_B_ as do (d) the softening temperature, *T*_s_, above which *G*′ > *G*′′ (not observed for PCL/B) and (e) *T*_d,rheo_. (f) In PCL-BTA/B, *T*_d,rheo_ corresponds to either *T*_d,NF_, for *w*_B_ ≤ 1.1 wt%, or *T*_d_ for *w*_B_ ≥ 1.5 wt% determined from DSC heating scans, but is slightly lower in PCL/B particularly at low B concentrations. Similar trends are also observed for *G*′ at 70 °C, *T*_s_, and *T*_d,rheo_ when plotted against the total concentration of BTA moieties, including the end groups, *w*_B__+__BTA_ (see ESI Fig. S16†). The dashed curves in all panels serve as guides to the eye.

The melt properties of the PCL-BTA/B blends are compared with those of the PCL/B reference blends based on oscillatory shear rheometry temperature sweeps at a frequency of 1 rad s^−1^ and a scanning rate of 10 °C min^−1^, in which the specimens were cooled from the homogeneous melt state (180–200 °C, depending on the B content) to 30 °C and heated back to the melt state. In what follows, we focus on the results for PCL-BTA/B obtained from the heating scans (Fig. 9, ESI Fig. S15†), which show one or more of the following features as the temperature is raised: (i) a steep drop in the storage modulus, *G*′, upon melting of the PCL matrix at its *T*_m_ of about 58 °C; (ii) a rubbery plateau at which *G*′ decreases slowly from a value in the range of 0.1–2.7 MPa (depending on the B content of the material, Fig. 9c); (iii) a crossover temperature referred to as the softening temperature, *T*_s_ (Fig. 9d), above which *G*′ < *G*′′, marking the upper limit of the rubbery plateau; (iv) a steep decrease in both *G*′ and *G*′′ towards a “rheological” dissociation temperature, *T*_d,rheo_ (Fig. 9e and f), above which *G*′ < 3.5 kPa and the behavior approaches that of a low-viscosity fluid (so that *G*′ falls below the sensitivity limit of the present setup).

In the PCL-BTA/B blends, *T*_d,rheo_ is generally close to either *T*_d,NF_, for *w*_B_ ≤ 1.1 wt%, or *T*_d_ for *w*_B_ ≥ 1.5 wt% (Fig. 9f), implying that the aggregates act as a reinforcement at temperatures *T* < *T*_d,rheo_. The absence of significant reinforcement between *T*_d,rheo_ and *T*_d_* suggests that the nanofibrils make a dominant contribution, where they are present, and that the structural reorganization that gives rise to *T*_d_* has no significant effect. In the PCL/B reference blends, *T*_d,rheo_ is generally somewhat lower than *T*_d_, but the two temperatures remain strongly correlated over the whole composition range. It follows that *T*_d,rheo_ decreases strongly with decreasing B concentration in both PCL-BTA/B and PCL/B (Fig. 9e).

Both PCL-BTA/B and PCL/B also show an increase in *G*′ with decreasing temperature towards a limiting value immediately above *T*_m_, which again decreases with decreasing B concentration, as reflected by 
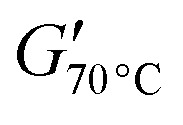
, the value of *G*′ measured at 70 °C (Fig. 9c). However, 
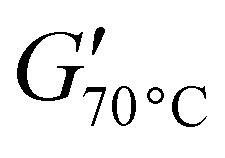
 is about six times higher in PCL-BTA/B than in PCL/B for a given *w*_B_, levelling off at about 2 MPa in PCL-BTA/B (4 wt%). Moreover, only PCL-BTA/B shows a true rubbery plateau delimited by *T*_m_ and *T*_s_; the latter is undefined for PCL/B, which remains a viscoelastic fluid (*G*′ < *G*′′) at all temperatures *T* > *T*_m_, regardless of the B concentration in the range investigated.

The additional rheological transition at *T*_s_ in the PCL-BTA/B modified blends, their rubbery elastic response in the temperature range *T*_m_ < *T* < *T*_s_, and their significantly increased *G*′ compared with the PCL/B reference blends are consistent with the formation of a network of polymer-bridged nanofibrils^94^ due to the incorporation of the PCL-BTA end groups in the B aggregates formed during cooling. This is strongly reminiscent of the behavior of the oligoalanine-based system described previously,^50^ but the transition temperatures *T*_d_, *T*_d,rheo_, and *T*_s_ are significantly higher in the PCL-BTA/B blends than in the oligoalanine-based materials, not least because the *T*_d_ value of the pure B additive is higher than that of the oligoalanine additive. The PCL-BTA/B materials are therefore expected to be processable by techniques that require a high melt strength and elasticity over a correspondingly wider temperature range.^60^ Moreover, thermal degradation of oligopeptides (decomposition temperature in air, *T*_dec_ ≈ 250 °C) limits their potential for high-temperature polymer processing, whereas the BTA-based materials show much greater stability (*T*_dec_ ≈ 350 °C).

**Fig. 10:**
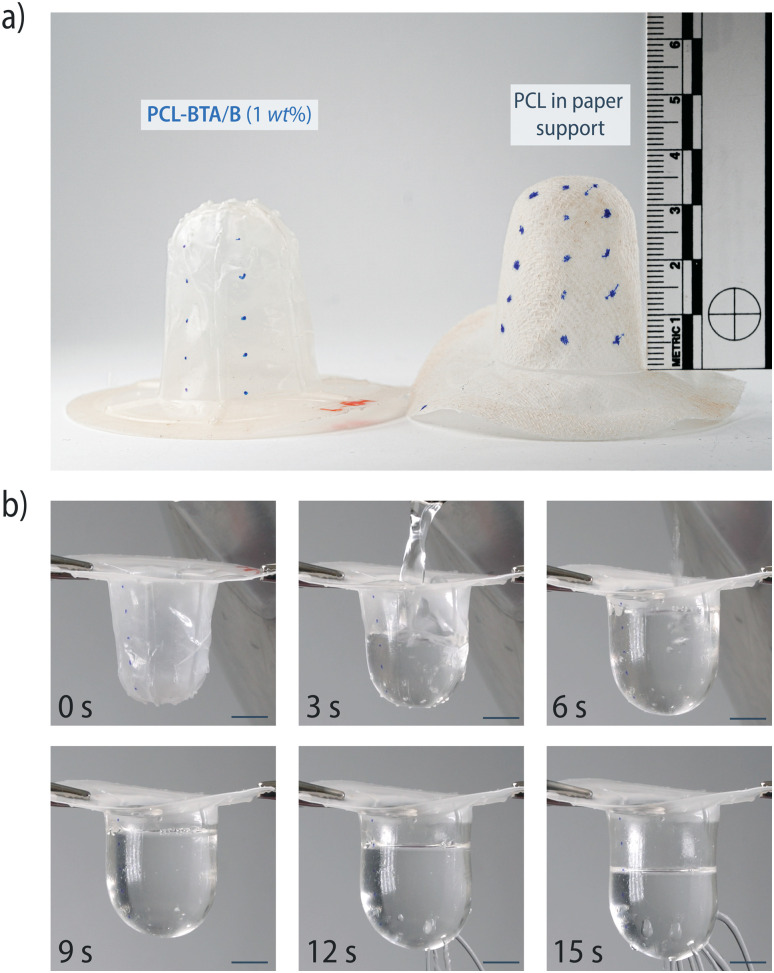
(a) PCL-BTA/B (1 wt%) has been processed into cup-shaped objects by thermoforming, whereas pristine PCL can only be thermoformed with the aid of a paper support. (b) The PCL-BTA/B cups were filled with boiling water. Defects associated with the connection of the mold to the vacuum pump during thermoforming resulted in water leakage after 12 s but the cups remained globally intact. Scale bars represent 1 cm.

To demonstrate the improved processability, we successfully processed PCL-BTA/B (1 wt%) films into cup-shaped specimens by thermoforming at 70 °C (Fig. 10); this is not possible for free-standing unmodified PCL films. When filled with boiling water, the PCL-BTA/B cups become transparent due to the loss of crystallinity in the PCL matrix at temperatures above *T*_m_ = 58 °C but retain their shape and support the weight of water, thanks to the elastic response of the nanofibrillar network at this temperature.

**Fig. 11:**
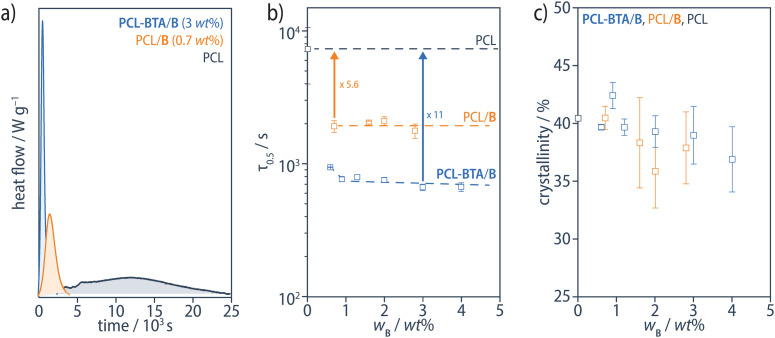
(a) Examples of isothermal DSC traces for PCL-BTA/B (blue), PCL/B (orange) and pristine PCL (grey) at 48 °C after cooling from 200 °C at 10 °C min^−1^ to 70 °C, followed by rapid cooling to 48 °C, from which (b) the crystallization half times, *τ*_1/2_, have been determined. *τ*_1/2_ is decreased by a factor of up to 5.6 in PCL/B (orange), and up to 11 in blends of PCL-BTA/B (blue) compared with pristine PCL (grey). The dashed curves serve as guides to the eye. (c) The degree of crystallinity of the PCL matrix, as determined by integrating the PCL melting peak in heating scans at 10 °C min^−1^ recorded immediately after the isothermal step, assuming the melting enthalpy of an ideal PCL crystal to be 139.5 J g^−1^.^95^

BTA derivatives are well known to be efficient nucleating agents for polymer crystallization. In iPP, they typically form microfibrous or needle-like crystalline bulk precipitates with high surface-to-volume ratios,^75^ similar to the structures observed in the PCL/B reference blends. The much higher surface-to-volume ratio areas of the nanofibrils formed in the PCL-BTA/B blends may further enhance their nucleation effect. To investigate this, we determined the crystallization half time (*τ*_1/2_) in isothermal DSC experiments at 48 °C after cooling from the melt. While *τ*_1/2_ is reduced by a factor of up to 5.6 in PCL/B reference blends with respect to pristine PCL, it is reduced by a factor of 11 in PCL-BTA/B, for all *w*_B_ ≥ 1 wt% (Fig. 11a and b). The resulting crystallinity, determined by integrating the PCL melting peak in heating scans at 10 °C min^−1^ immediately after the isothermal step and assuming the melting enthalpy of an ideal PCL crystal to be 139.5 J g^−1^,^95^ is around 40% at *w*_B_ ≤ 2 wt% but decreases somewhat at higher B concentrations (Fig. 11c). Hence, the nanofibrils in the PCL-BTA/B materials are confirmed to nucleate PCL crystallization more effectively than the bulk additive. This may be due to their high degree of dispersion and/or the covalent attachment of the polymer chains.^96^

To emphasize the importance of using high molar mass polymers, we also studied BTA-end-modified PCL of *M̄*_n_ = 24 900 g mol^−1^ (PCL25-BTA), with an end-group concentration of 3.2 wt%. There is no evidence from DSC, FTIR, or shear rheology experiments for end-group aggregation in PCL25-BTA in the absence of B, consistent with previous reports on BTA-modified PCL.^59^ However, PCL25-BTA/B blends contain co-assembled nanoscopic aggregates similar to those in PCL-BTA/B. Moreover, due to its higher end-group concentration, the threshold for the onset of bulk B precipitation increases to *w*_B_ ≥ 3 wt% in PCL25-BTA, indicating that more B participates in the formation of the nanoscopic aggregates (ESI Fig. S17†). In agreement with our previously reported findings for low-*M̄*_n_ PCL modified with oligo-l-alanine end groups,^50^PCL25-BTA/B remains brittle (ESI Fig. S17f†) despite the presence of the reinforcing network, because the molar mass of the base polymer is too low for the establishment of well-developed, stable entanglements.

## Conclusions

We have shown that high-molar-mass PCL-BTA, modified with BTA-based self-assembling end groups, undergoes nanophase separation when blended with the low-molar-mass BTA derivative B in the low-concentration regime, *w*_B_ ≤ 1.1 wt%. In this regime, we observe the quantitative formation of strongly hydrogen-bonded nanoscopic aggregates by co-assembly of the end groups and additive. This is particularly remarkable considering not only the high molar mass of the PCL but also its polarity, which has been reported to render supramolecular network formation difficult, even for substantially lower molar masses.^59^ Accordingly, pure PCL-BTA shows no evidence of end-group assembly. Moreover, the bulk B precipitates seen in unmodified PCL/B reference blends in this concentration range are absent from PCL-BTA/B. Indeed, weakly hydrogen-bonded colloidal B domains persist in PCL-BTA/B at temperatures well above the dissociation temperature of the aggregates, presumably stabilized by the PCL-BTA end groups. This may impede the bulk precipitation of B upon cooling, instead providing precursors for the formation of well-defined, polymer-bridged, high-aspect-ratio BTA nanofibrils.

In the bulk melt state, the resulting physically crosslinked, reinforced polymer network manifests itself as a rubbery plateau in rheometry temperature sweeps. Depending on the B content, this plateau extends up to about 90 °C above the PCL melting point and is associated with a storage modulus more than two orders of magnitude higher than that of pristine PCL or PCL-BTA in this temperature range, and some six times higher than that of PCL/B containing the same amount of B. The resulting materials show significantly enhanced thermal stability and facilitate processing by thermoforming, for instance. In addition, the polymer-tethered BTA nanofibrils are far more efficient nucleating agents for polymer crystallization than the corresponding bulk B precipitates, with the potential to reduce industrial process cycle times and hence manufacturing costs. Moreover, a comparison with blends using modified PCL of lower molar mass demonstrates that it is precisely the combination of a well-entangled base polymer and the polymer-bridged nanofibril network that renders PCL-BTA/B technologically interesting. Finally, the concept of end-group and additive co-assembly may in principle be extended to the many other aliphatic polyesters currently considered to be more sustainable alternatives to conventional plastics, but which often lack sufficient melt strength for certain key processing techniques, thus broadening manufacturing options.

## Materials and methods

### Materials

The following chemicals were obtained from commercial sources and used without further purification: PCL, *M̄*_n_ = 80 000 g mol^−1^ (Sigma), triethylamine ≥ 99.5% (Sigma), *N*,*N*′-dicyclohexylcarbodiimide (DCC) 99% (Sigma), 3-methylbutylamine ≥ 98% (Sigma), 1,3,5-benzenetricarbonyl trichloride 98% (Sigma), *N*,*N*-diisopropylethylamine (DIPEA) 99% (abcr), trimethyl benzene-1,3,5-tricarboxylate 98% (abcr), 4-dimethyl aminopyridine (DMAP, Fluorochem), 1*H*-benzotriazol-1-yloxytripyrrolidinophosphoniumhexafluorophosphate (PyBOP, Fluorochem), *N*-(9-fluorenylmethoxycarbonyl)-glycine (GlyFmoc) ≥98% (novabiochem), piperidine 99% (Acros Organics), DMSO-d_6_ 99.8% D (ZEOtope), CDCl_3_ 99.8% D, stabilized with Ag (ZEOtope), diethyl ether (Reactolab), ethanol (EtOH, Reactolab), hexane (Reactolab), ethyl acetate (EtOAc, Reactolab), argon 99.999% (Carbagas), HCl 25% (Sigma), KOH (Reactolab), NaOH (Reactolab), LiOH ≥98% (Fisher Chemicals). Tetrahydrofuran (THF), methanol (MeOH), and CHCl_3_ (Reactolab) were distilled prior to use. Dichloromethane (DCM) ≥99.9% stabilized with amylene (Sigma) was distilled prior to use.

### Synthetic procedures

#### *N*,*N*′,*N*′′-Tris(isopentyl)-1,3,5-benzenetricarboxamide (B)

3-Methylbutylamine (0.785 g, 9.0 mmol), triethylamine (5.08 g, 50.0 mmol), and THF (150 mL) were added to a flask at 0 °C. 1,3,5-Benzenetricarbonyl trichloride (0.664 g, 2.5 mmol) was added slowly to the reaction mixture over 10 min, and the mixture was stirred at 0 °C for 1 h and then at room temperature for 16 h. The resulting white precipitate was filtered and discarded, the volume of the remaining solution was reduced to about 50 mL under reduced pressure, and the solution was poured into water (500 mL). The resulting white solid product was collected, washed with 0.5 M KOH (25 mL) and water (50 mL), and dried to give a white powder (0.84 g, 81%). ^1^H NMR (DMSO-*d*_6_, 400 MHz, 64 scans): *δ*/ppm = 8.62 (t, 3H), 8.35 (s, 3H), 3.30 (q, 6H), 1.63 (m, 3H), 1.45 (q, 6H), 0.92 (d, 18H). ^13^C NMR (100 MHz, CDCl_3_): *δ*/ppm = 166.46, 135.72, 127.83, 38.79, 38.58, 26.12, 22.59. HRMS (nanochip-ESI/LTQ-Orbitrap) *m*/*z*: [M + H]^+^ calcd for C_24_H_40_N_3_O_3_^+^ 418.3064; found: 418.3058. EA: calcd for C_24_H_39_N_3_O_3_: C, 69.03; H, 9.41; N, 10.06. Found: C, 67.96; H, 9.28; N, 9.78. For ^1^H NMR and ^13^C NMR spectra, see ESI Figs. S18–S20.†

#### 5-Methoxycarbonylbenzene-1,3-dicarboxylic acid (1)

Trimethyl benzene-1,3,5-tricarboxylate (20.2 g, 80 mmol) was added to MeOH (200 mL) in a 1 L round-bottom flask. The mixture was stirred to create a slurry. Sodium hydroxide (7.04 g 176 mmol) was dissolved in MeOH (400 mL) and then added to the slurry. The mixture was heated to reflux to obtain a clear solution. After 16 h, the volume of the solution was reduced to about 300 mL by rotary evaporation, and it was poured into 1 m HCl (1 L). The solution was then extracted with diethyl ether (4 × 200 mL). The combined organic phase was washed once with 1 m HCl (125 mL), and the solvent was subsequently removed *in vacuo* to give a white powder (18.0 g) that was refluxed in CHCl_3_ (460 mL) and EtOH (4.4 mL) for 15 min, followed by hot filtration. The filter cake was collected and the refluxing procedure was repeated twice more to give crude 1 as a white powder (16.5 g, 92%), which was used without further purification. ^1^H NMR (400 MHz, DMSO-*d*_6_): *δ*/ppm = 8.66 (s, 1H), 8.64 (s, 2H), 3.93 (s, 3H). For ^1^H NMR spectra, see ESI Fig. S21.†

#### Methyl 3,5-bis(isopentylcarbamoyl)benzoate (2)

Crude product 1 (7.0 g, 31.2 mmol), 3-methylbutylamine (6.80 g, 78.0 mmol), PyBOP (40.6 g, 78 mmol), and THF (300 mL) were combined in a 500 mL round-bottomed flask. DIPEA (20 g, 156 mmol) was added to the mixture, and the mixture was stirred at room temperature. After 16 h, the volume was reduced to 75 mL under reduced pressure and the remaining solution was precipitated into water (1 L). The precipitate was collected, redissolved in MeOH (50 mL), and precipitated into water again (700 mL). The precipitate was collected and dried to yield crude product 2 (10.5 g, 93%, containing approximately 12 mol% B), which was used without further purification. However, for analysis, 2 was purified by column chromatography using hexane/EtOAc (7 : 3) as the eluent. ^1^H NMR (400 MHz, DMSO-*d*_6_): *δ*/ppm = 8.76 (t, 2H), 8.55 (d, 1H), 8.53 (d, 2H), 3.93 (s, 3H), 3.32 (m, 4H), 1.63 (m, 2H), 1.45 (q, 4H), 0.92 (d, 12H). ^13^C NMR (100 MHz, DMSO- *d*_6_): *δ*/ppm = 161.03, 160.95, 130.77, 126.22, 125.62, 124.98, 47.87, 33.92, 33.64, 22.16, 17.71. HRMS (nanochip-ESI/LTQ-Orbitrap) *m*/*z*: [M + H]^+^ calcd for C_20_H_30_N_2_O_4_^+^ 363.2206; found: 363.2274. For HRMS, ^1^H NMR and ^13^C NMR spectra, see ESI Figs. S22–S24.†

#### *N*,*N*′-Bis(isopentylcarbamoyl)benzoic acid (3)

Crude product 2 (10.5 g, 29.0 mmol) was dissolved in MeOH (200 mL), and the resulting mixture was filtered to remove any insoluble material. LiOH (1.80 g, 75.0 mmol) was dissolved in a mixture of water (15 mL) and MeOH (50 mL), and the resulting solution was added to the MeOH solution. This reaction mixture was stirred for 16 h at room temperature. The mixture was then acidified with 1 m HCl (≈ 150 mL) until pH ≈ 2 was reached. The resulting white precipitate was collected and dried to give crude 3 as a white powder (9.5 g). The crude product was then added to CHCl_3_ (200 mL). The resulting suspension was refluxed for 10 min under vigorous stirring, allowed to cool to room temperature, and filtered. This process was repeated twice more, until TLC indicated that the side product tris(3-methylbutyl) benzene-1,3,5-tricarboxamide (*R*_f_ = 0.3, heptane/EtOAc, 3 : 2) had been completely removed. Pure 3 was obtained as a white powder (8.0 g, 80%). ^1^H NMR (400 MHz, toluene-*d*_8_, 375 K): *δ*/ppm = 12.72 (br, 1H), 8.82 (s, 2H), 8.75 (s, 1H), 8.09 (br, 2H), 3.43 (q, *J* = 6.9 Hz, 4H), 1.63 (m, 2H), 1.50 (q, *J* = 6.9 Hz, 4H), 0.88 (d, *J* = 6.5 Hz, 12H). ^13^C NMR (100 MHz, DMSO-*d*_6_): *δ*/ppm = 166.52, 164.86, 135.34, 131.10, 130.35, 130.08, 37.99, 37.60, 25.24, 22.41. HRMS (nanochip-ESI/LTQ-Orbitrap) *m*/*z*: [M + H]^+^ calcd for C_19_H_29_N_2_O_4_^+^ 349.2122; found 349.2118. EA: calcd for C_19_H_28_N_2_O_4_: C, 65.49; H, 8.10; N, 8.04. Found: C, 65.31; H, 8.10; N, 8.01. For HRMS, ^1^H NMR, and ^13^C NMR spectra, see ESI Figs. S25–S27.†

#### PCL

To obtain a reference ^1^H NMR spectrum of non-modified commercial poly(ε-caprolactone) (PCL), 1 g of the polymer was dissolved in DCM (3 mL) and precipitated into MeOH (25 mL). This process was repeated twice more, and the purified PCL was dried under high vacuum. The number-average molar mass, *M̄*_n_ = 112 000 g mol^−1^, was determined by GPC (polystyrene calibration). The number-average molar mass, *M̄*_n_ = 103 000 g mol^−1^, was also calculated from the ratio of C**H**_**2**_–OH end group signals to all PCL backbone peak integrals, implying an end group content of ≥92% OH end groups. ^1^H NMR (400 MHz, CDCl_3_, 512 scans): *δ*/ppm = 4.04 (1817 H, t, C**H**_2_OCO_PCL_), 3.63 (4 H, t, C**H**_**2**_–OH), 2.29 (1813 H, t, C**H**_**2**_COO_PCL_), 1.68 (3714 H, m, C**H**_**2,PCL**_), 1.37 (1813 H, m, C**H**_**2,PCL**_), GPC: *M̄*_n_ = 112 000, *M̄*_w_ = 164 000, *Đ* = 1.47. GPC traces are shown in ESI Fig. S28.†

#### PCL-GlyFmoc

PCL and *N*-(9-fluorenylmethoxycarbonyl)-glycine (GlyFmoc) were dried under high vacuum prior to use. GlyFmoc (1.041 g, 3.5 mmol, 8.8 equiv.), DMAP (0.293 g, 2.4 mmol, 6.0 equiv.), and DCC (1.465 g, 7.1 mmol, 17.8 equiv.) were dissolved in DCM (280 mL) under an argon atmosphere. Dry PCL (40 g, 0.4 mmol, 1 equiv.) was then added and the colorless reaction mixture was stirred at room temperature for 6 d, during which time it turned yellow. The mixture was precipitated into MeOH (2 L). The precipitate was redissolved in DCM (280 mL), and reprecipitated into MeOH (2 L) twice more. The precipitate was collected and dried under vacuum at 30 °C to yield PCL-GlyFmoc as a colorless solid (39.6 g, 99%). ^1^H NMR (400 MHz, CDCl_3_, 512 scans): *δ*/ppm = 7.77 (4H, d, C**H**_**ar**_), 7.59 (4H, d, C**H**_**ar**_), 7.40 (4H, t, C**H**_**ar**_), 7.31 (4H, t, C**H**_**ar**_), 6.48–5.34 (2H, 3 s, N**H**), 4.39 (4H, d, CH–C**H**_**2**_–NH), 4.16 (t, OCO–C**H**_**2**_–NH), 4.03 (1524H, t, C**H**_2_OCO_PCL_), 3.93 (t, C**H**_**Fmoc**_), 2.31 (1529H, t, C**H**_**2**_COO_PCL_), 1.68 (3185H, m, C**H**_**2,PCL**_), 1.41 (1561H, m, C**H**_**2,PCL**_).

#### PCL-GlyNH_2_

PCL-GlyFmoc (39.6 g, 0.4 mmol, 1 equiv.) was dissolved in DCM (280 mL) under an argon atmosphere, and piperidine (0.35 mL, 3.5 mmol, 8.8 equiv.) was added. The reaction mixture was stirred at room temperature, more piperidine (0.35 mL, 3.5 mmol, 8.8 equiv.) was added after 1 d, and stirring was continued for 2 d. The reaction mixture was precipitated into MeOH (2 L). The precipitate was redissolved in DCM (280 mL) and again precipitated into MeOH (2 L) twice more. The precipitate was washed with MeOH and dried under vacuum at 30 °C to yield PCL-GlyNH_2_ as a colorless solid (35 g, 88%). ^1^H NMR (400 MHz, CDCl_3_, 512 scans): *δ*/ppm = 4.05 (1500H, t, C**H**_**2**_OCO_PCL_), 2.30 (1564H, t, C**H**_**2**_COO_PCL_), 1.64 (3175H, m, C**H**_**2**,PCL_), 1.38 (1472H, m, C**H**_**2**,PCL_).

#### PCL-BTA

*N*,*N*′-Bis(isopentylcarbamoyl)benzoic acid 3 (0.3356 g, 0.96 mmol, 2.9 equiv.), DIPEA (0.21 mL, 1.2 mmol, 3.6 equiv.), and PyBOP (0.546 g, 1.0 mmol, 3.0 equiv.) were dissolved in DCM (280 mL) under an argon atmosphere. PCL-GlyNH_2_ (35 g, 0.33 mmol, 1 equiv.) was added to the slightly turbid mixture. The reaction mixture was stirred at room temperature for 3 d and then precipitated into MeOH (1.5 L). The precipitate was dissolved in DCM (280 mL), and re-precipitated into MeOH (1.5 L) twice more. The precipitate was collected and dried under vacuum at 30 °C to yield PCL-BTA as a white solid (34.2 g, 98%). ^1^H NMR (400 MHz, CDCl_3_, 512 scans): *δ*/ppm = 8.36 (6H, s, C**H**_**ar**_), 7.04–6.46 (3H, 3s, N**H**), 4.05 (2068H, t, C**H**_**2**_OCO_PCL_), 3.48 (9H, m, C**H**_**2**_–NH), 2.30 (2057H, t, C**H**_**2**_COO_PCL_), 1.65 (4418H, m, C**H**_**2**,PCL_), 1.37 (2153H, m, C**H**_**2**,PCL_), 0.96 (31H, d, C**H**_**3**_). GPC: *M̄*_n_ = 107 000, *M̄*_w_ = 166 000, *Đ* = 1.54. GPC traces are shown in ESI Fig. S28.†

#### PCL25-GlyFmoc

PCL and *N*-(9-fluorenylmethoxycarbonyl)-glycine (GlyFmoc) were dried under high vacuum prior to use. GlyFmoc (1.57 g, 5.28 mmol, 8.8 equiv.), DMAP (0.440 g, 3.6 mmol, 6.0 equiv.), and DCC (2.204 g, 10.68 mmol, 17.8 equiv.) were dissolved in DCM (100 mL) under an argon atmosphere. Dry PCL (15 g, 0.6 mmol, 1 equiv.) was then added and the colorless reaction mixture was stirred at room temperature for 3 d, during which time it turned yellow. The mixture was precipitated into MeOH (0.7 L). The precipitate was redissolved in DCM (100 mL) and reprecipitated into MeOH (0.7 L) twice more. The precipitate was collected and dried under vacuum at 30 °C to yield PCL25-GlyFmoc as a colorless solid (14 g, 93%). ^1^H NMR (400 MHz, CDCl_3_, 512 scans): *δ*/ppm = 7.75 (4H, d, C**H**_**ar**_), 7.59 (4H, d, C**H**_**ar**_), 7.40 (4H, t, C**H**_**ar**_), 7.31 (4H, t, C**H**_**ar**_), 5.34 (2H, s, N**H**), 4.41 (4H, d, CH–C**H**_**2**_–NH), 4.16 (t, OCO–C**H**_**2**_–NH), 4.05 (205H, t, C**H**_2_OCO_PCL_), 3.87 (t, C**H**_**Fmoc**_), 2.30 (189H, t, C**H**_**2**_COO_PCL_), 1.64 (395H, m, C**H**_**2,PCL**_), 1.38 (216H, m, C**H**_**2,PCL**_). For the ^1^H NMR spectrum, see ESI Fig. S29.†

#### PCL25-GlyNH_2_

PCL-GlyFmoc (14 g, 0.56 mmol, 1 equiv.) was dissolved in DCM (100 mL) under an argon atmosphere and piperidine (0.49 mL, 4.9 mmol, 8.8 equiv.) was added. The reaction mixture was stirred at room temperature, more piperidine (0.49 mL, 4.9 mmol, 8.8 equiv.) was added after 1 d, and stirring was continued for 2 d. The reaction mixture was precipitated into MeOH (0.7 L). The precipitate was redissolved in DCM (100 mL) and again precipitated into MeOH (0.7 L) twice more. The precipitate was washed with MeOH and dried under vacuum at 30 °C to yield PCL25-GlyNH_2_ as a colorless solid (10 g, 71%). ^1^H NMR (400 MHz, CDCl_3_, 512 scans): *δ*/ppm = 8.72 (4H, s, N**H**_**2**_), 4.05 (382H, t, C**H**_**2**_OCO_PCL_), 2.30 (364H, t, C**H**_**2**_COO_PCL_), 1.64 (750H, m, C**H**_**2**,PCL_), 1.37 (364H, m, C**H**_**2**,PCL_). For the ^1^H NMR spectrum, see ESI Fig. S30.†

#### PCL25-BTA

*N*,*N*′-Bis(isopentylcarbamoyl)benzoic acid 3 (0.4055 g, 1.26 mmol, 2.9 equiv.), DIPEA (0.252 mL, 1.44 mmol, 3.6 equiv.), and PyBOP (0.6552 g, 1.2 mmol, 3.0 equiv.) were dissolved in DCM (70 mL) under an argon atmosphere. PCL25-GlyNH_2_ (10 g, 0.40 mmol, 1 equiv.) was added to the slightly turbid mixture. The reaction mixture was stirred at room temperature for 1 d and then precipitated into MeOH (0.4 L). The precipitate was dissolved in DCM (70 mL) and re-precipitated into MeOH (0.4 L) twice more. The precipitate was collected and dried under vacuum at 30 °C to yield PCL25-BTA as a white solid (8.5 g, 85%). ^1^H NMR (400 MHz, CDCl_3_, 512 scans): *δ*/ppm = 8.32 (6H, s, C**H**_**ar**_), 7.37–6.46 (4H, 3s, N**H**), 4.05 (533H, t, C**H**_**2**_OCO_PCL_), 3.86 (5H, m, C**H**_**2**_–NH), 2.29 (525H, t, C**H**_**2**_COO_PCL_), 1.63 (1108H, m, C**H**_**2**,PCL_), 1.37 (527H, m, C**H**_**2**,PCL_), 0.96 (27H, d, C**H**_**3**_). GPC: *M̄*_n_ = 24 900, *M̄*_w_ = 33 100, *Đ* = 1.33. GPC traces are shown in ESI Fig. S28.† For the ^1^H NMR spectrum, see ESI Fig. S31.†

### Methods

#### Blend preparation

PCL-BTA or pristine PCL were blended with B by dissolving the respective mixture of compounds in hot THF and stirring the mixture for 15 min after complete dissolution. THF was then evaporated under reduced pressure and the blends dried under high vacuum until further use. Unless mentioned otherwise, we compare PCL/B and PCL-BTA/B blends with equal *w*_B_ in wt%. For the corresponding [B] in mol L^−1^, the combined *w*_B__+__BTA_ for the additive and end groups in wt%, [B + BTA] in mol L^−1^, and the additive-to-end-group ratio, [B]/[BTA], in mol mol^−1^, see Table 1.

#### Differential scanning calorimetry (DSC)

Temperature-dependent DSC measurements were performed using a Mettler Toledo DSC 3+ instrument under constant nitrogen flow (50 mL min^−1^) with a default scanning rate of 10 °C min^−1^. For the PCL-BTA/B and PCL/B blends, specimens of 20–30 mg were subjected to three heating and two cooling scans to check for reproducibility. Only the linear baseline-corrected second heating and first cooling scans were considered. Isothermal DSC experiments were conducted by heating the specimens to the homogeneous melt state (up to 180 °C depending on the composition) and then cooling them to the required measurement temperature and maintaining this temperature until the end of the crystallization process.

#### Fourier transform infrared spectroscopy (FTIR)

Specimens for FTIR spectroscopy in attenuated total reflectance (ATR) mode were prepared in the differential scanning calorimeter by cooling a few mg of the material from the homogeneous melt state to room temperature at 10 °C min^−1^. Specimens for temperature-dependent FTIR spectroscopy in transmission mode were prepared by drop-casting PCL-BTA/B (1 mg mL^−1^ in THF) onto Real Crystal® KBr sample cards, and cooling the film from the melt state (180 °C) to room temperature at a nominal rate of 10 °C min^−1^. All IR spectra were recorded with a JASCO FT/IR 6300 spectrometer using the Miracle ATR accessory from PIKE. Peak deconvolution of the transmission spectra in selected regions was performed using the Peak® spectroscopy software. Deconvolution was performed in the spectral region between 3000 and 3500 cm^−1^ based on three peaks: one for the N–H stretching vibration at 3235 cm^−1^, one for the N–H stretching vibration at 3396 cm^−1^, and one for the sharp absorption at 3437 cm^−1^ assigned to water absorbed by PCL.

#### Optical microscopy (OM)

Specimens of a few mg were compressed between glass cover slides after heating to the homogeneous melt state (up to 220 °C depending on the composition) and then cooled at a nominal rate of 10 °C min^−1^ to the desired observation temperature using a Linkam TMS600 hot-stage. Images were recorded using a Sony Alpha6700 digital camera mounted on an Olympus BH2 optical microscope with an LMscope adaptor in either bright field or between cross-polarizers.

#### Atomic force microscopy (AFM)

Selected materials were cooled from the melt at 200 °C to ambient temperature at 10 °C min^−1^. Flat surfaces were prepared by hot-pressing the specimens directly onto a steel AFM stub at the desired temperature (between the PCL matrix melting point and the dissociation temperature of the fibrils) with the aid of an amorphous Kapton™ H release film (DuPont) using a Linkam TMS600 hot stage. They were then cooled to room temperature at 10 °C min^−1^ and the release film was removed. Images were recorded using an Asylum Research Cypher VRS AFM equipped with a heating stage and a laser-excited MikroMasch aluminum-coated NC14 probe (resonance frequency 160 kHz, force constant 5 N m^−1^, tip radius of curvature < 7 nm) in intermittent contact mode, with a typical scanning frequency of 5 Hz and amplitude ratios of 0.3–0.5. Images were analyzed using the NIH software ImageJ.

#### Interfibrillar distance calculation

An idealized interfibrillar distance, *d*_NF_, was calculated as described elsewhere for oligoalanine-modified PCL blended with a matching oligoalanine molecule,^50^ assuming a uniform dispersion of hexagonally close-packed nanofibrils composed of a single stack of B and PCL-BTA end groups,3
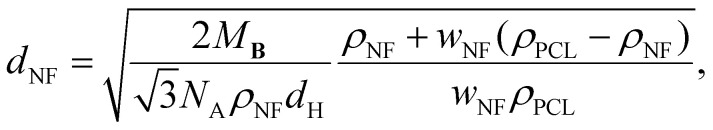
where *M*_B_ = 417.6 g mol^−1^ is the molar mass of the additive, *N*_A_ is Avogadro's number, and *d*_H_ = 3.5 Å is the intermolecular spacing along the nanofibril axis,^89^*ρ*_NF_ = 1.11 g cm^−3^ is the density of the nanofibril taken from single crystal analysis of B,^98^*w*_NF_ is the nanofibril weight fraction and is approximated by the sum of the additive weight fraction, *w*_B_, and the end-group weight fraction, *w*_BTA_, and *ρ*_PCL_ = 1.06 g cm^−3^ is the melt density of the PCL matrix.^99^

#### Shear rheometry

Dynamic shear rheometry was performed using a parallel plate TA Instruments ARES 2 rheometer. 8 mm diameter steel plates were used throughout, with the gap set to 0.5 mm. The blend specimens were loaded in their homogeneous melt state (at up to 230 °C depending on the composition) and tested at a nominal rate of 10 °C min^−1^ with a fixed radial frequency of 1 rad s^−1^ and a strain of 0.1%.

#### Small- and wide-angle X-ray scattering (SAXS/WAXS)

Specimens for SAXS and WAXS analysis were prepared in the rheometer in the open parallel plate configuration by cooling the material from the melt state (up to 230 °C depending on the composition) to room temperature at a nominal rate of 10 °C min^−1^. 2D patterns were collected over a time period of 1 min under vacuum using a Xenocs Xeuss 3.0 instrument with a GeniX3D system. A Cu K_α_ (*λ* = 1.54 Å) micro source was used in transmission mode with a Dectris detector at a distance of 200 mm. The intensity profiles were obtained by radial integration of the 2D patterns using XSACT software from Xeuss.

#### Hot-pressing

The specimens were placed in an aluminum mold with dimensions of 25 × 25 × 0.03 cm^3^ and a square hole (5 × 5 cm^2^) in the center, which was then placed between Teflon plates with lateral dimensions of 50 × 50 cm^2^. A Lauffer Pressen UVL 5.0 laboratory press was loaded at 25 °C and evacuated to 1 mbar before heating to 190 °C at a nominal rate of 10 °C min^−1^. After the melt temperature was reached, a pressure of 60 N m^−1^ was applied; the temperature was held for 1 min, followed by cooling to ambient temperature at a nominal rate of 10 °C min^−1^ and subsequent release of the pressure.

#### Thermoforming

Circular hot-pressed specimens with a diameter of *d* = 7 cm and a thickness of 0.3 mm were processed into a cup (30 mm in diameter and 35 mm in depth) above the melting temperature of PCL using a Formech HD686 vacuum former by heating for 20–30 s in an infrared oven, and thermoforming after a delay of 0.4 s, with 100% plug assist, 50% vacuum power, 0% air pressure and 0% pre-stretch into a tempered 40 °C mold. Circular defects resulted from the application of vacuum through the holes in the steel mold, which was in direct contact with PCL-BTA/B. To thermoform PCL, a paper support was added to the mold prior to the addition of the polymer melt.

#### Thermal dimensional stability testing

To test the dimensional stability of the thermoformed cup-shaped specimens at elevated temperatures, they were filled with boiling water from a household kettle and a video recording was used for analysis.

#### Gel permeation chromatography (GPC)

The number-average molar mass, *M̄*_n_, the weight-average molar mass, *M̄*_w_, and the dispersity, *Đ*, were determined by dissolving a 3–5 mg sample in 1 mL THF and filtering the solution through a 0.220 μm PTFE filter before injection. Elution was performed in THF at 40 °C at a flow rate of 1 mL min^−1^ using an Agilent 1260 Infinity instrument incorporating the 390-MDS detector train equipped with a refractive index detector, one PSS SDV precolumn and either two PLgel 5 μm MIXED-C Analytical columns or two PSS SDV Analytical Linear XL columns. Calibration was performed with polystyrene standards with *M̄*_n_ in the range of 682–2 520 000 g mol^−1^.

#### 1D NMR spectroscopy

^1^H NMR spectra were obtained at 25 °C using a Bruker Avance III 400 spectrometer at a frequency of 400 MHz and calibrated with respect to the residual solvent peak of CHCl_3_ (7.26 ppm) or DMSO (2.50 ppm). Chemical shifts are expressed in parts per million (ppm) (s = singlet, d = doublet, t = triplet, m = multiplet).

#### Flory–Huggins fitting for PCL/B

The concentration-dependent *T*_d_ obtained from DSC heating scans of PCL/B was fitted to the Flory–Huggins model, assuming the Flory–Huggins interaction parameter was that given by eqn (1). Because the molar volume of PCL is much greater than that of the additive, the variation of *T*_d_ with the additive volume fraction, *ϕ*, is given by4
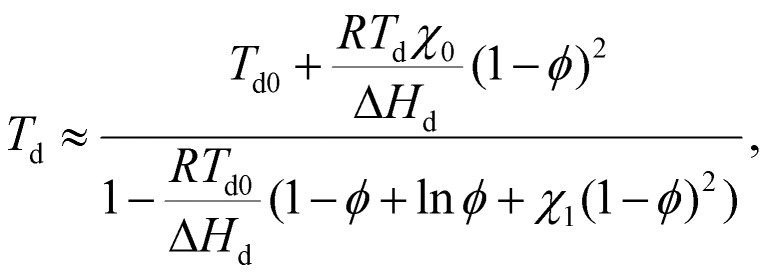
where Δ*H*_d_ = 31 kJ mol^−1^ and *T*_d0_ are the molar enthalpy of dissociation and the dissociation temperature, respectively, of the pure additive. Fitting eqn (4) to the observed *T*_d_ for the PCL/B blends gives *χ*_0_ = 1633 K and *χ*_1_ = −2.569.

## Author contributions

H.F. and D.G. developed the research concept and designed the experiments. S.T. carried out the DSC, FTIR, OM, XRD, and rheology experiments and analyzed the data. M.G. developed the synthesis procedure for the BTA-based reagent for polymer end modification and additive B. M.W. performed the temperature-dependent FTIR measurements and the Flory–Huggins analysis. C.J.G.P. carried out the AFM experiments. S.T., D.G., C.J.G.P., and H.F. wrote the manuscript.

## Conflicts of interest

The authors declare no conflict of interests.

## Supplementary Material

QO-012-D5QO00087D-s001

## Data Availability

The authors declare that the data supporting the findings of this study are available within the paper and its ESI.†
