# Supplementary material for: Polymer-bridged nanofibrils in a high-molar-mass polyester via co-assembly of benzenetricarboxamide end groups and an additive
Source: Org Chem Front. 2025 May 13;12(20):5395–413. doi: 10.1039/d5qo00087d (PMC12142735; doi:10.1039/d5qo00087d)
Supplement: QO-012-D5QO00087D-s001 [file QO-012-D5QO00087D-s001.pdf]

Supplementary Information (SI) for Organic Chemistry Frontiers.
This journal is © the Partner Organisations 2025

Polymer-Bridged Nanofibrils in a High-Molar-Mass Polyester via Co-assembly of Benzenetricarboxamide End Groups and Additive

Supporting Information

*Sophia Thiele[1], Michael Giffin, Matthieu Wendling[1], Daniel Görl[1], Christopher J. G. Plummer[1], Holger Frauenrath[1]**

[1]Ecole Polytechnique Fédérale de Lausanne (EPFL)

Institute of Materials

1015 Lausanne, Switzerland

* Corresponding Author :

holger.frauenrath@epfl.ch

Supplementary Figures

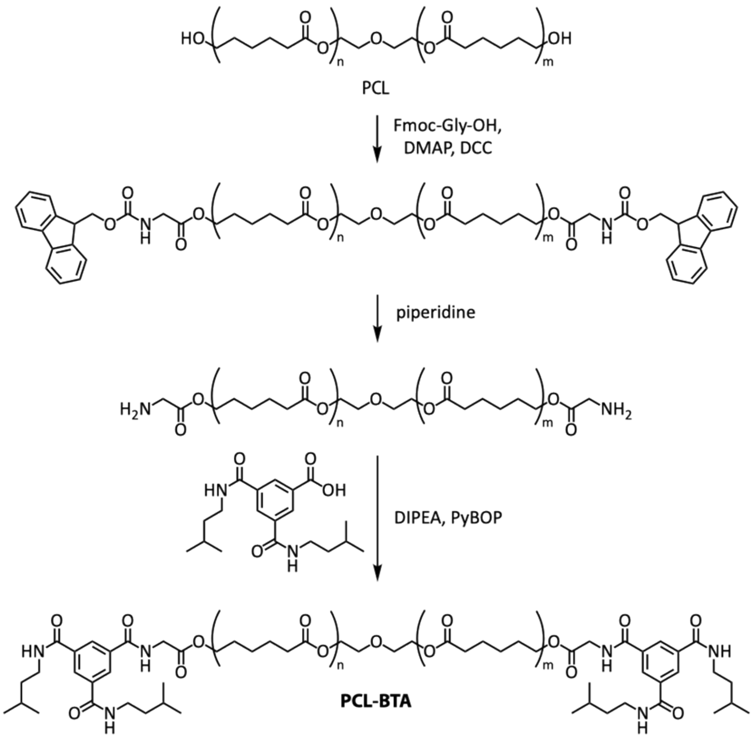

Supplementary Scheme 1. End modification of commercial telechelic PCL in three steps to give **PCL-BTA**.

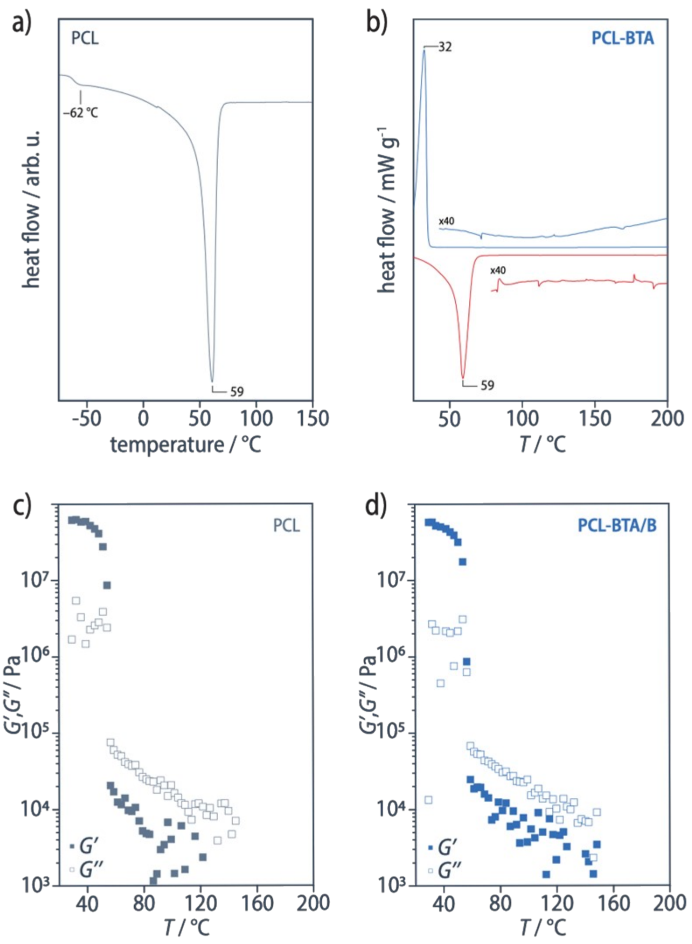

Supplementary Figure S1. *a)* Second DSC heating scan of pristine PCL at 10 °C/min showing the glass transition temperature, T_g = –62 °C, and the melting peak at T_m = 58 °C. *b)* Oscillatory shear rheometry heating scan of pristine PCL at 10 °C/min, showing the storage modulus, G', and loss modulus, G'', which are consistent with a low viscosity polymer melt at temperatures above the PCL melting peak at T_m = 58 °C. *c)* Second DSC heating scan (red) and first DSC cooling scan (blue) of **PCL-BTA** at 10°C/min showing no thermal transitions indicative of end group aggregation. *d)* Oscillatory shear rheometry heating scan of end-modified **PCL-BTA** closely resemble those of pristine PCL, consistent with an absence of end-group aggregation under these conditions.

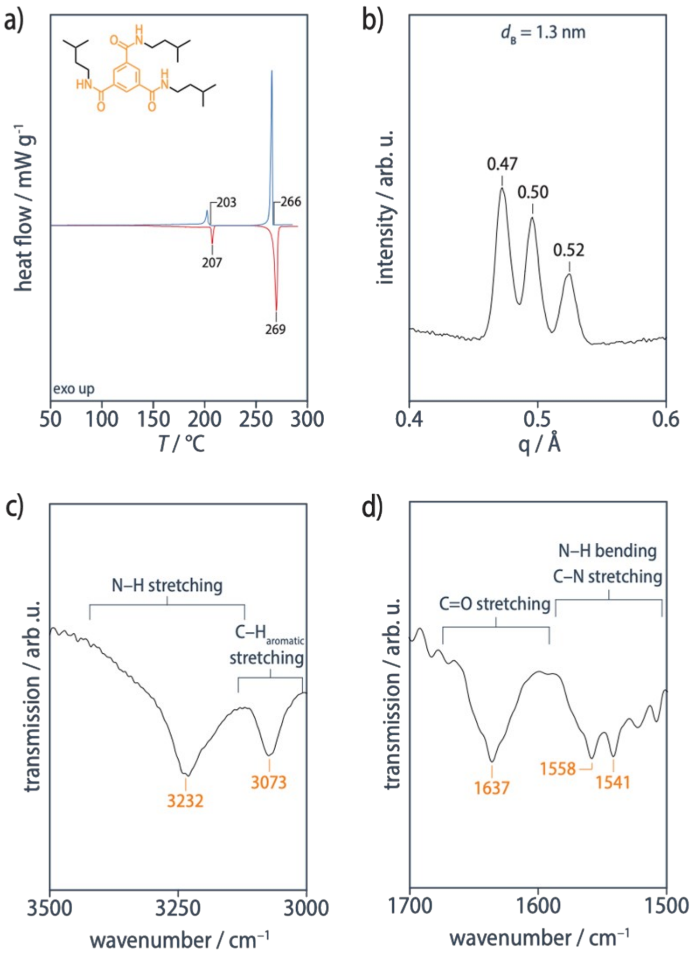

Supplementary Figure S2. *a)* DSC second heating (red) and first cooling (blue) scans of **B** at 10 °C/min. Upon heating, **B** undergoes a first thermal transition from the bulk crystalline state to a mesophase at T_{meso} = 207 °C, followed by a second transition to the homogeneous melt at the dissociation temperature, T_{d} = 269 °C. Upon cooling, aggregation occurs at T_{agg} = 266 °C to form the mesophase, which crystallizes at $T_{\text{c,}\mathbf{B}}$ = 203 °C. *b)* Bragg reflections in SAXS patterns of pure **B** recorded at 25 °C. *c)* FTIR spectra recorded at ambient temperature show the N–H stretching band at 3232 cm^{-1}, *d)* the C=O stretching band at 1637 cm^{-1}, and the combined N–H bending/C–N stretching band at 1558 cm^{-1} and 1541 cm^{-1}, all of which are consistent with strongly hydrogen-bonded aggregates of **B**.[1]

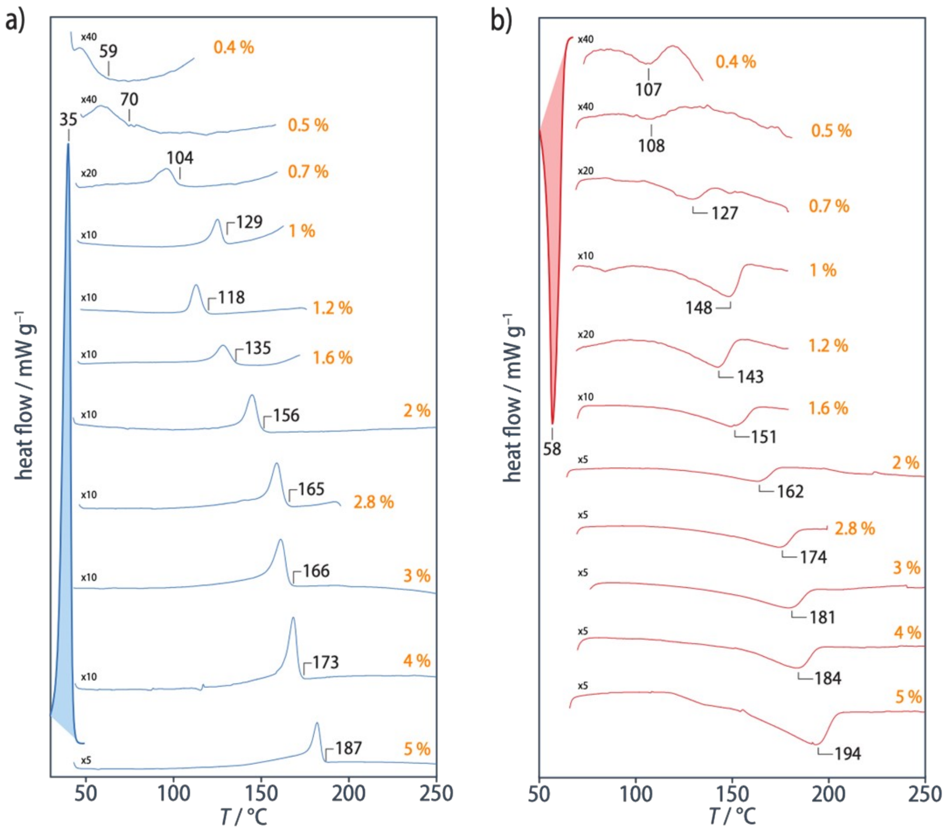

Supplementary Figure S3. *a)* First DSC cooling scans of PCL/**B** at −10 °C/min show an exothermic transition at temperatures above the nominal crystallization temperature of PCL at $T_c \approx 35$ °C (shown only for one composition for clarity) identified with the association temperature of **B** aggregates, T_{agg}. *b)* Second heating scans at a scanning rate of 10 °C/min show the melting endotherm of PCL at $T_m = 58$ °C (shown only for one composition for clarity) and an additional endothermic transition attributed to dissociation of the **B** aggregates. This latter shifts to higher temperatures as the **B** concentration increases.

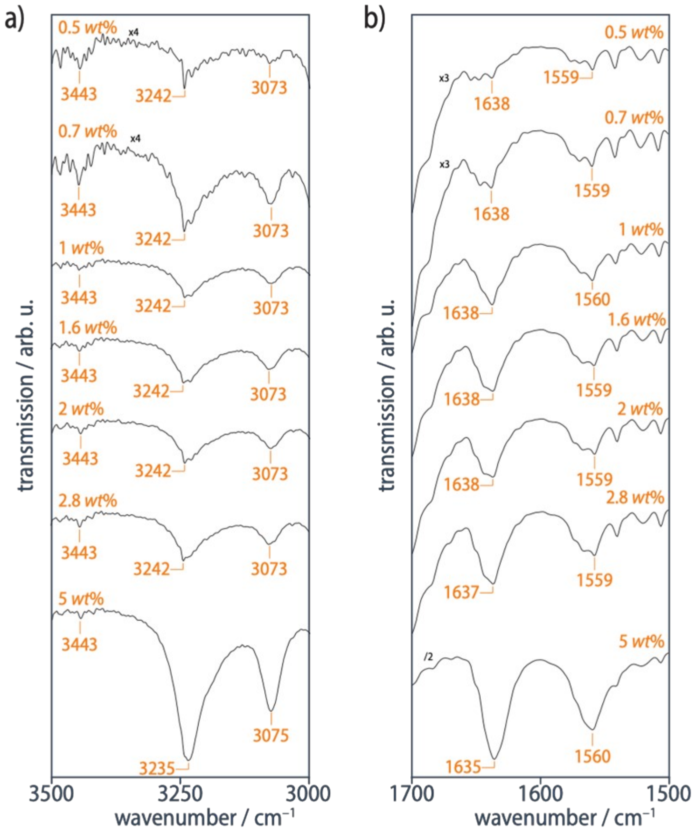

Supplementary Figure S4. ATR-FTIR spectra of PCL/**B** recorded at room temperature showing *a)* the N–H band (3240 cm^{-1}) and the C-$H_{aromatic}$ band (3075 cm^{-1}), and *b)* the C=O stretching (1638 cm^{-1}), and the combined N–H bending/C–N stretching band (1560 cm^{-1}). Bands at these positions are characteristic of strongly hydrogen-bonded, columnar **B** aggregates.[2]

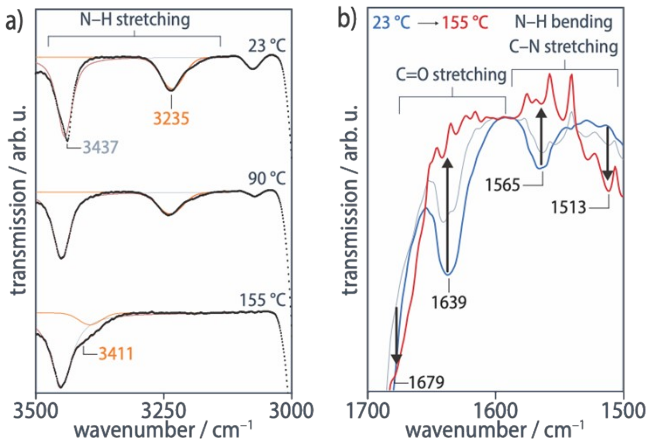

Supplementary Figure S5. FTIR transmission spectra of a PCL/**B** (1.2 *wt*%) film on KBr, recorded at 23, 90, and 155 °C, where *a)* the N–H stretching band at 3235 cm^{-1} indicative of strong BTA aggregates is visible at 23 and 90 °C, but shifts to 3411 cm^{-1} at 155 °C, indicating the presence of free amides. The experimental spectra (black) may be deconvoluted (red) into a peak for the N–H stretching band (orange) and a peak corresponding to the sharp band visible in all spectra at 3437 cm^{-1} (grey), which is assigned to water absorption by the PCL. *b)* The C=O stretching band and the combined N–H bending/C–N bands are again consistent with strongly hydrogen-bonded BTA at 23 and 90 °C, and free amides at 155 °C.

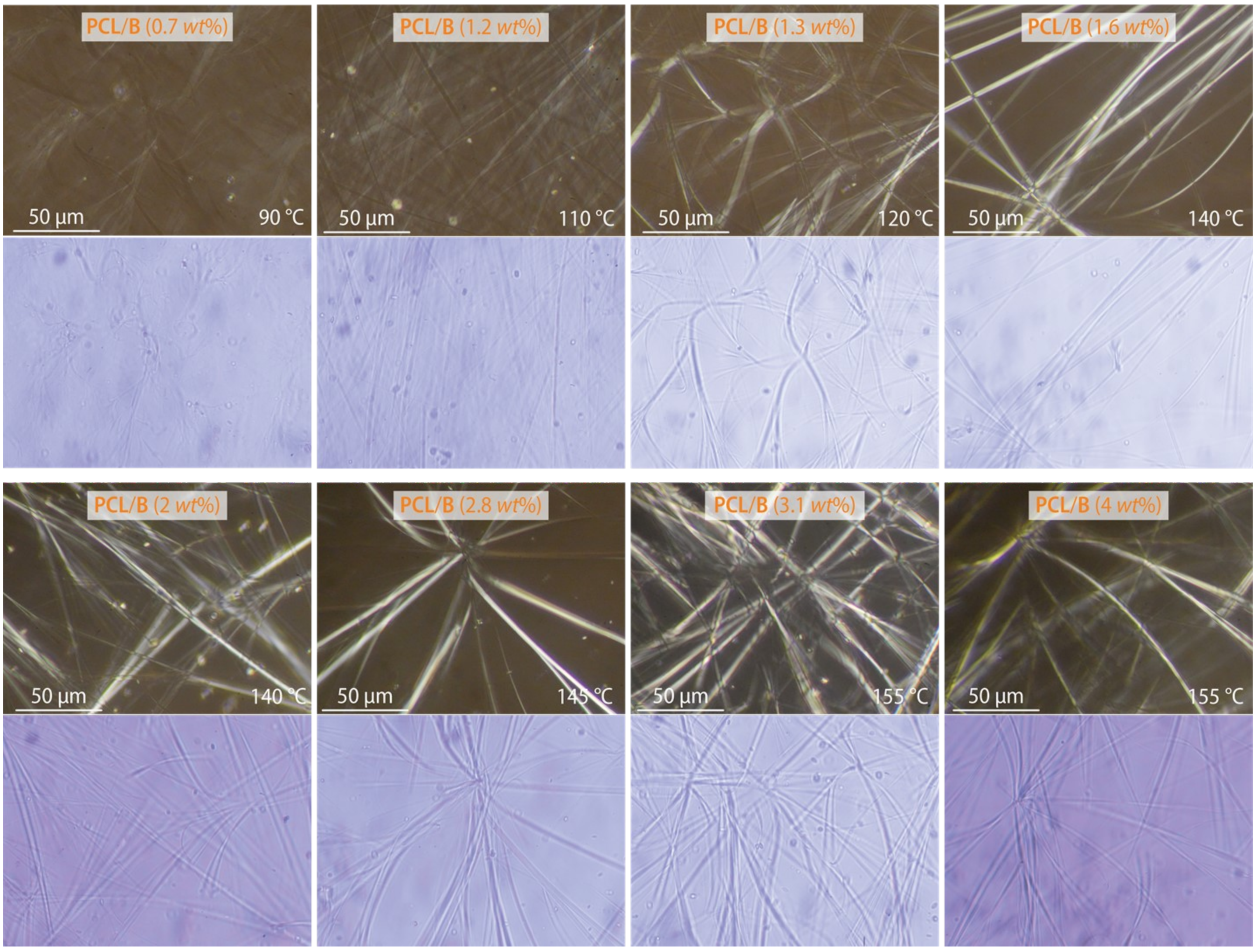

Supplementary Figure S6. Cross-polarized (top) and bright-field (bottom) optical microscopy of PCL/**B** at different **B** concentrations recorded while cooling at –10 °C/min from the isotropic melt state show characteristic needle-like crystals at temperatures between the exothermic transition at T_{agg} associated with **B** aggregation in DSC cooling curves and the crystallization temperature of the PCL matrix at about 35 °C.

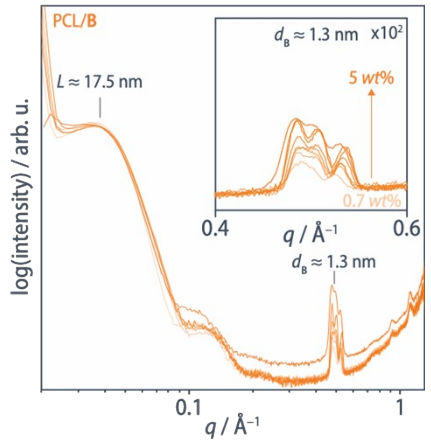

Supplementary Figure S7. 1D SAXS diffractograms from blends of PCL/**B**, measured on flat specimens obtained by cooling from the melt at –10 °C/min, scaled to the maximum intensity of the intense low q Bragg peaks corresponding to the lamellar long period, L, of the PCL matrix at 17–18 nm. The Bragg reflections in the neighborhood of $q \approx 0.24$ Å^{-1} ($d_B \approx$ 1.3 nm) correspond to macroscopic precipitates of **B**.

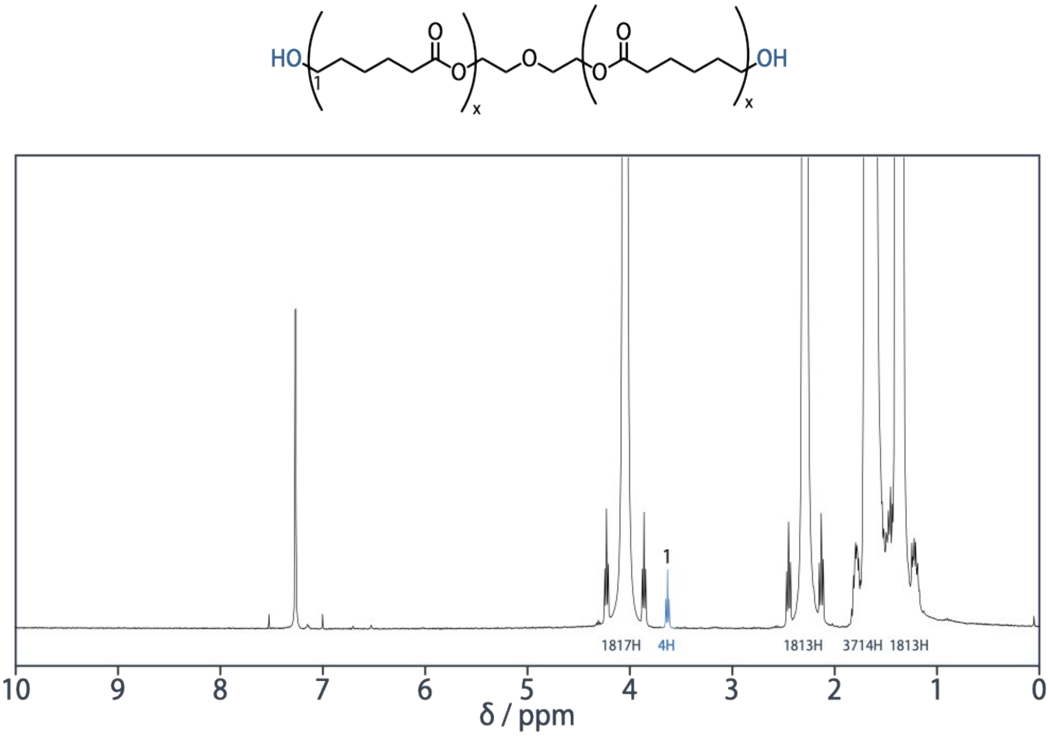

Supplementary Figure S8. In the ^{1}H NMR spectrum of PCL (400 MHz, $CDCl_3$, 512 scans), the integrals of peak **1** at δ = 3.63 ppm (4 H, t, C$\mathbf{H_2}$-OH) relative to those of the PCL backbone peaks at δ = 4.04 ppm (1817 H, t, C$\mathbf{H}_2$OCO$_{PCL}$), 2.29 ppm (1813 H, t, C$\mathbf{H_2}$COO$_{PCL}$), 1.68 ppm (3714 H, m, C$\mathbf{H_{2,PCL}}$), and 1.37 ppm (1813 H, m, C$\mathbf{H_{2,PCL}}$) indicate a number-average molar mass $\bar{M}_n$ = 104'000 g/mol as compared with $\bar{M}_n$ = 112'000 g/mol determined by GPC, from which a degree of (di-) functionalization f = 92% OH end groups is determined.

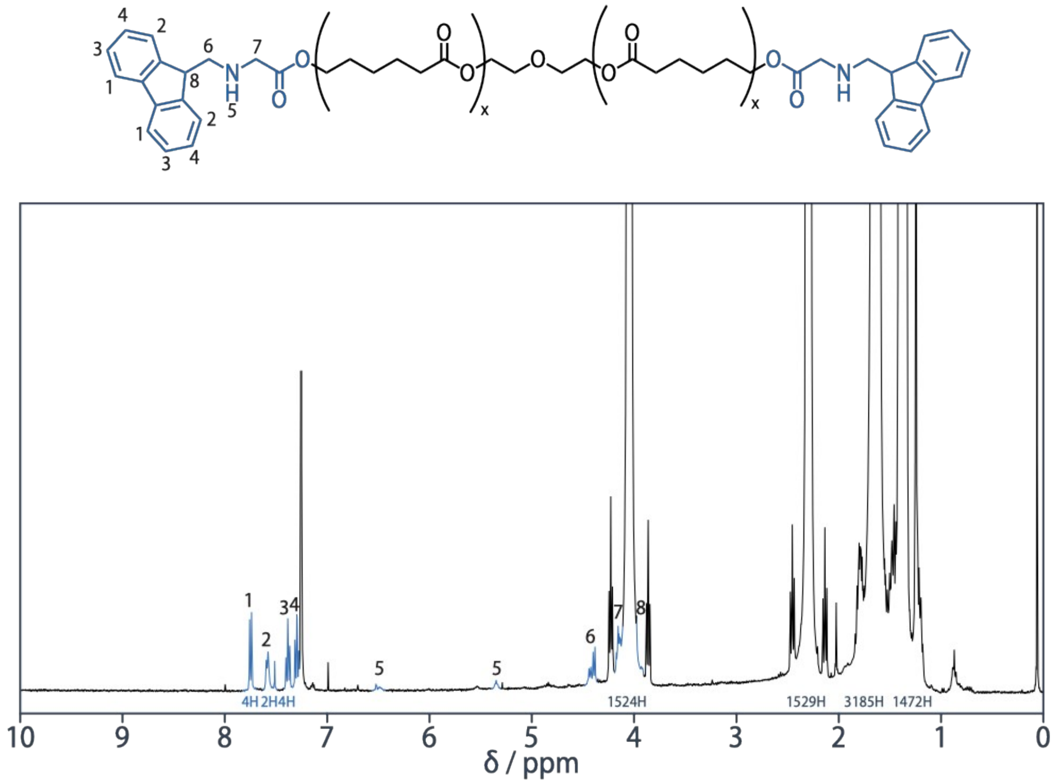

Supplementary Figure S9. ^{1}H NMR spectra of **PCL-GlyFmoc** (400 MHz, $CDCl_3$, 512 scans) show peaks at δ = 7.77 ppm (4H, d, C$\mathbf{H}_{\mathbf{ar}}$), 7.59 ppm (4H, d, C$\mathbf{H}_{\mathbf{ar}}$), 7.40 ppm (4H, t, C$\mathbf{H}_{\mathbf{ar}}$), 7.31 ppm (4H, t, C$\mathbf{H}_{\mathbf{ar}}$), 6.48-5.34 ppm (2H, 3 s, N**H**), 4.39 ppm (4H, d, CH-C$\mathbf{H_2}$-NH), 4.16 ppm (t, OCO-C$\mathbf{H_2}$-NH), 4.03 ppm (1524H, t, C$\mathbf{H}_2$OCO$_{PCL}$), 3.93 ppm (t, C$\mathbf{H}_{\mathbf{Fmoc}}$), 2.31 ppm (1529H, t, C$\mathbf{H_2}COO_{PCL}$), 1.68 ppm (3185H, m, C$\mathbf{H}_{\mathbf{2,PCL}}$), 1.41 ppm (1561H, m, C$\mathbf{H}_{\mathbf{2,PCL}}$), while peak **1** corresponding to C$\mathbf{H_2}$-OH observed in PCL is absent, indicating quantitative conversion of PCL to **PCL-GlyFmoc**.

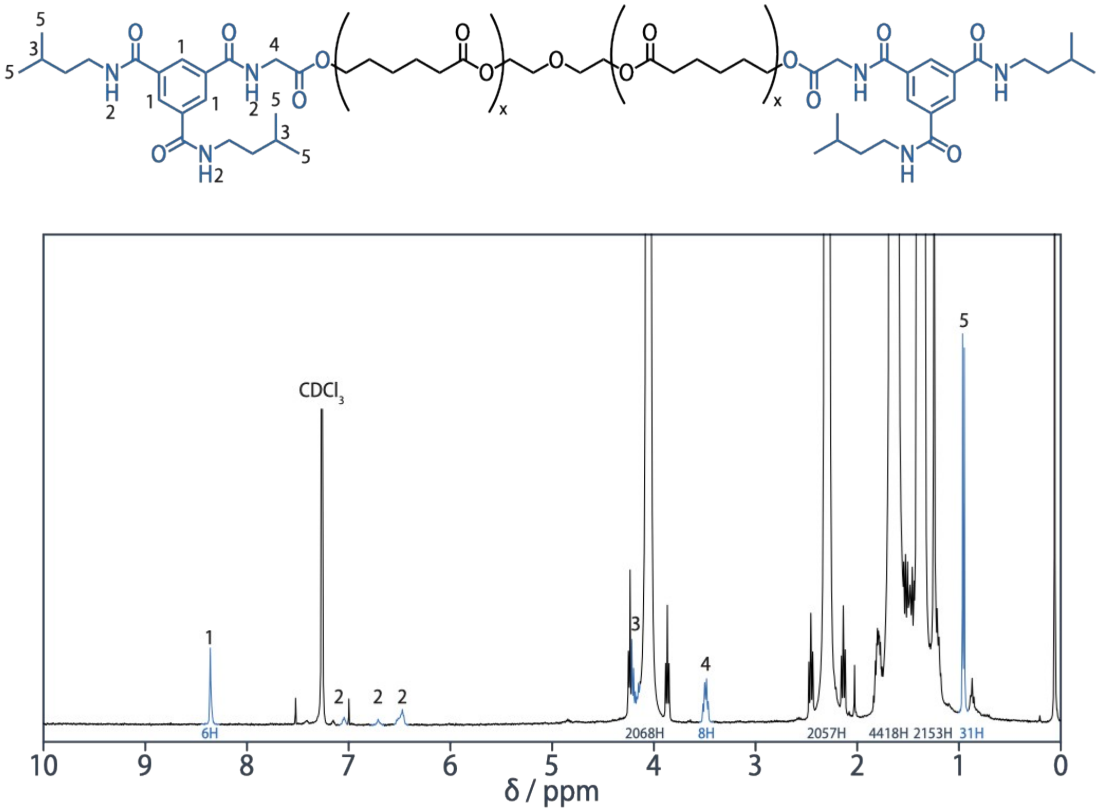

Supplementary Figure S10. ^{1}H-NMR spectra of **PCL-BTA** (400 MHz, $CDCl_3$, 512 scans) show peaks at δ = 8.36 ppm (6H, s, C**H$_{ar}$**), 7.04–6.46 ppm (3H, 3s, N**H**), 4.05 (2068H, t, C**H$_2$**OCO$_{PCL}$), 3.48 ppm (9H, m, C**H$_2$**-NH), 2.30 ppm (2057H, t, C**H$_2$**COO$_{PCL}$), 1.65 ppm (4418H, m, C**H$_{2,PCL}$**), 1.37 ppm (2153H, m, C**H$_{2,PCL}$**), 0.96 ppm (31H, d, C**H$_3$**), indicating quantitative end-group conversion to **PCL-BTA**.

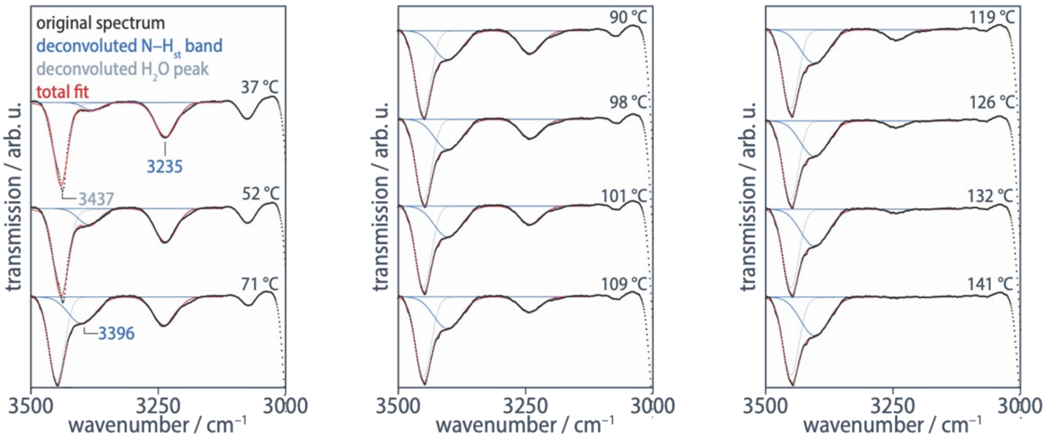

Supplementary Figure S11. Temperature-dependent FTIR transmission spectra recorded at 37–141 °C on a **PCL-BTA/B** (1 *wt*%) film on KBr show the N–H stretching band at 3235 cm^{-1} indicative of strong BTA aggregates at low temperature, but which shifts to 3396 cm^{-1} at elevated temperatures, indicating weakly hydrogen bonding BTA. The experimental spectra (black) may be deconvoluted (red), into a peak for the N–H stretching band (blue) and a peak corresponding to the sharp band visible in all spectra at 3437 cm^{-1} (grey), which is assigned to water absorption by the PCL.

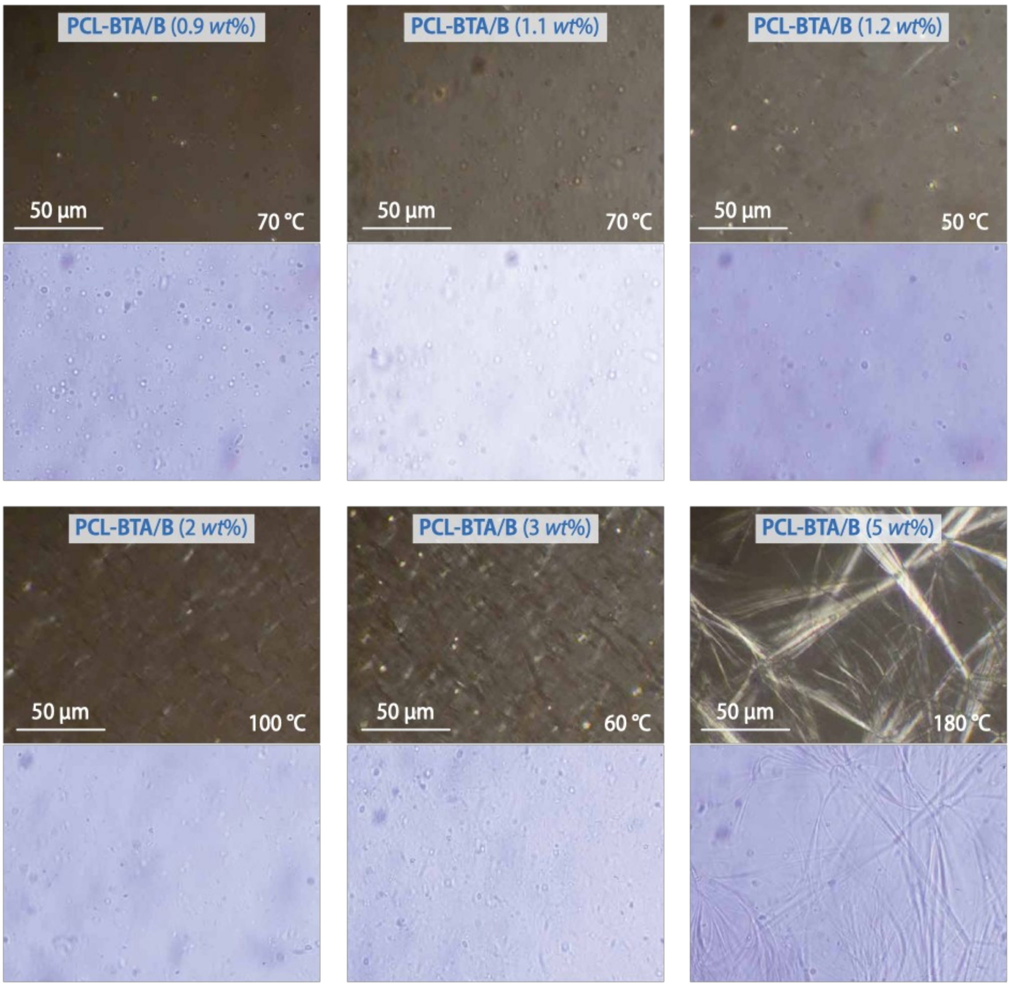

Supplementary Figure S12. Cross-polarized (top) and bright-field (bottom) optical micrographs of **PCL-BTA/B** recorded at $T < T_{agg}$ during cooling at 10 °C/min from the melt state. **PCL-BTA/B** blends with [**B**] ≤ 1.1 *wt*% remain optically homogeneous, whereas blends with [**B**] ≥ 1.2 *wt*% show microfibrous precipitates or needle-like crystals at higher concentrations.

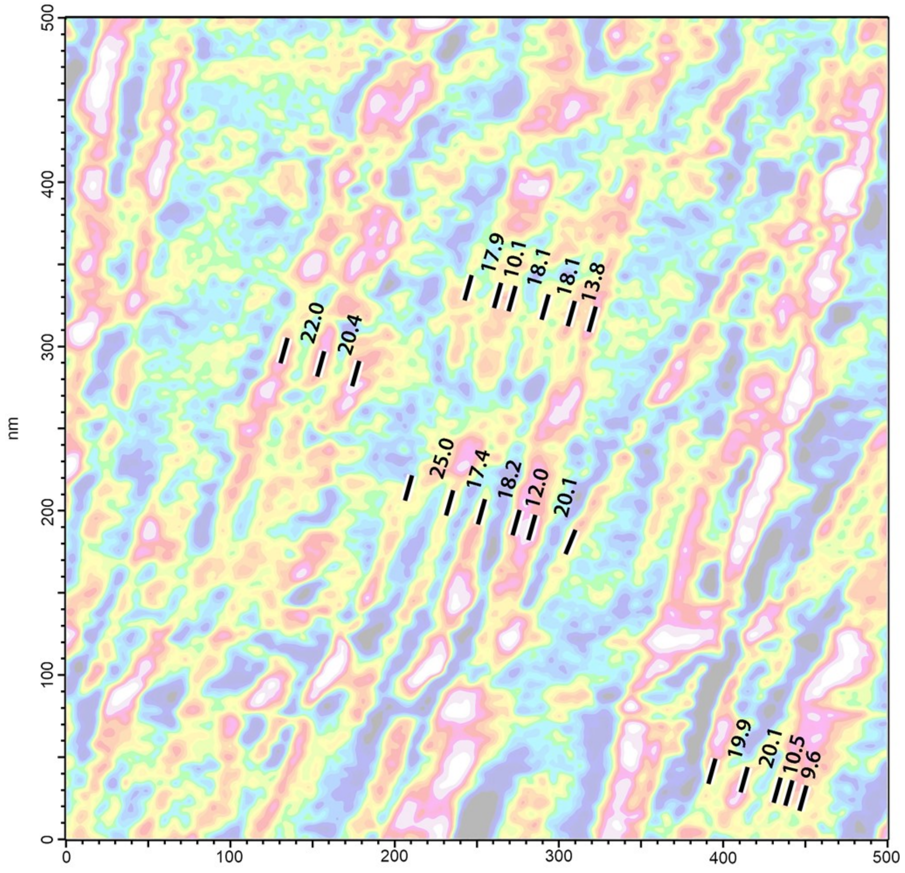

Supplementary Figure S13. AFM phase image of **PCL-BTA/B** (0.6 *wt*%) recorded at 70 °C after application of a band-pass filter, using false colors to highlight the positions of the nanofibrils. The distances between the nanofibrils indicated in the figure were determined from the peak separations in grey level line scans taken perpendicular to the fibrillar trajectories.

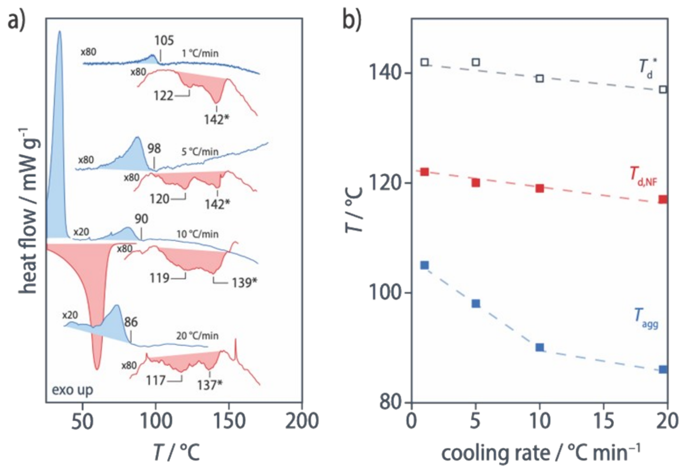

Supplementary Figure S14. *a)* DSC cooling scans (blue) of **PCL-BTA/B** (1.1 *wt*%), recorded at various scanning rates and subsequent heating curves (red) recorded at 10 °C/min. *b)* The positions of the endothermic transitions observed in the heating scans and associated with dissociation of nanofibrils of **B**, $T_{\mathrm{d,NF}}$, and with the melting of reorganized **B** at the higher temperature, T_{d}^{*}, depend little on the prior cooling rate. By contrast, the exothermic transition associated with **B** aggregation during cooling, T_{agg}, is rate-dependent and approaches $T_{\mathrm{d,NF}}$ as the cooling rate is decreased.

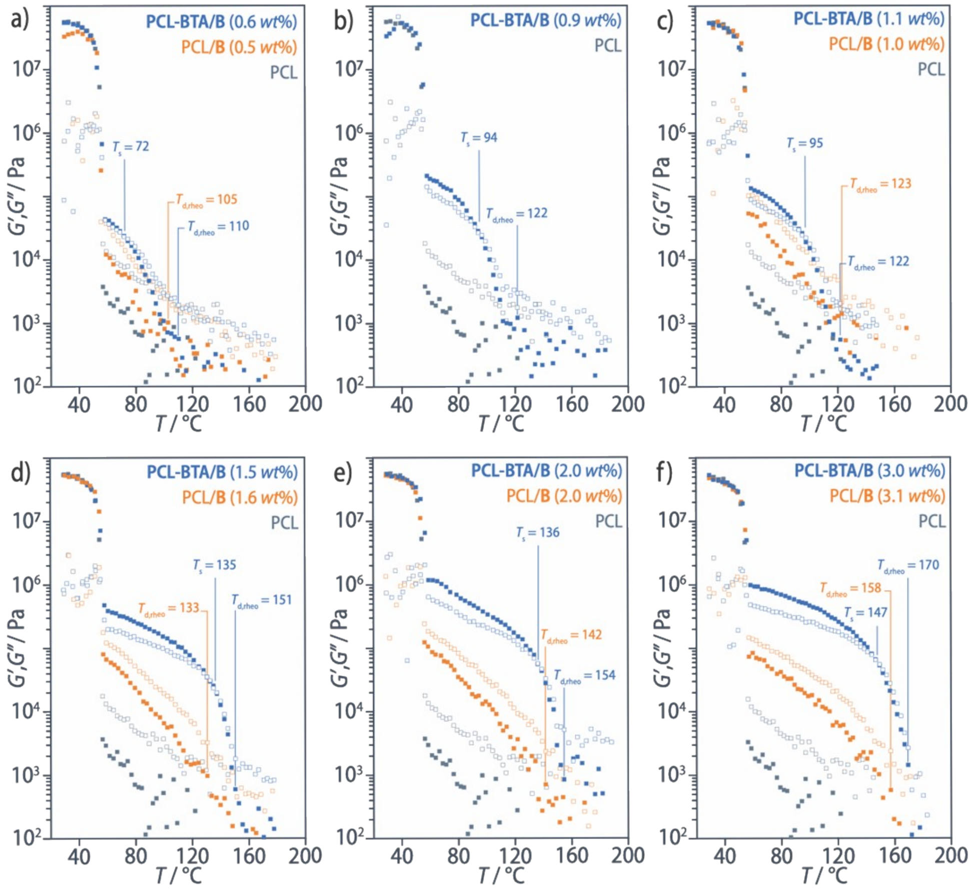

Supplementary Figure S15. Plots of storage (G') and loss (G'') moduli from oscillatory shear rheometry heating scans (10 °C/min, 0.1 % strain, at 1 rad/s) on **PCL-BTA/B** blends (blue) and PCL/**B** blends (orange) with various **B** concentrations.

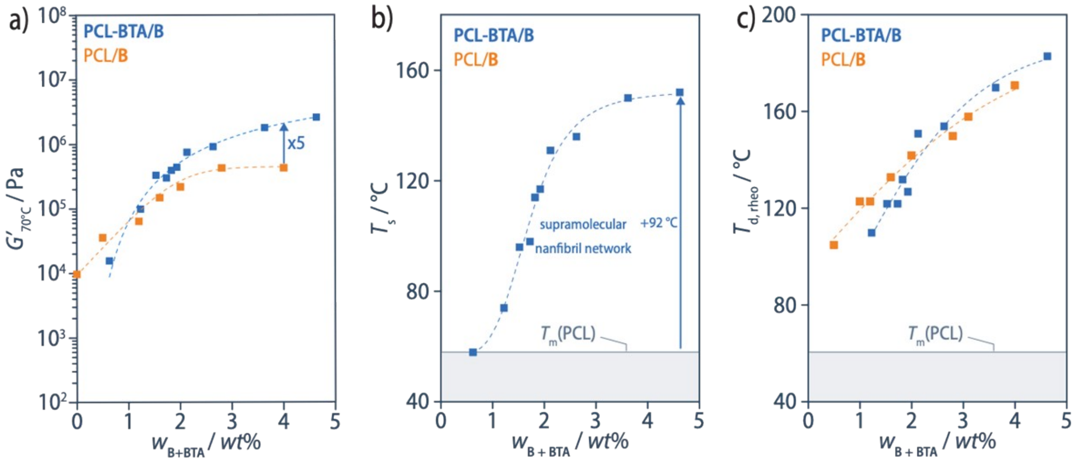

Supplementary Figure S16. *a)* G' at 70 °C increases systematically with the total concentration of BTA moieties, including the end groups, $w_{\mathbf{B+BTA}}$, as do *b)* the softening temperature, T_s, above which $G' > G''$ (not observed for PCL/**B**) and *c)* $T_{d,rheo}$.

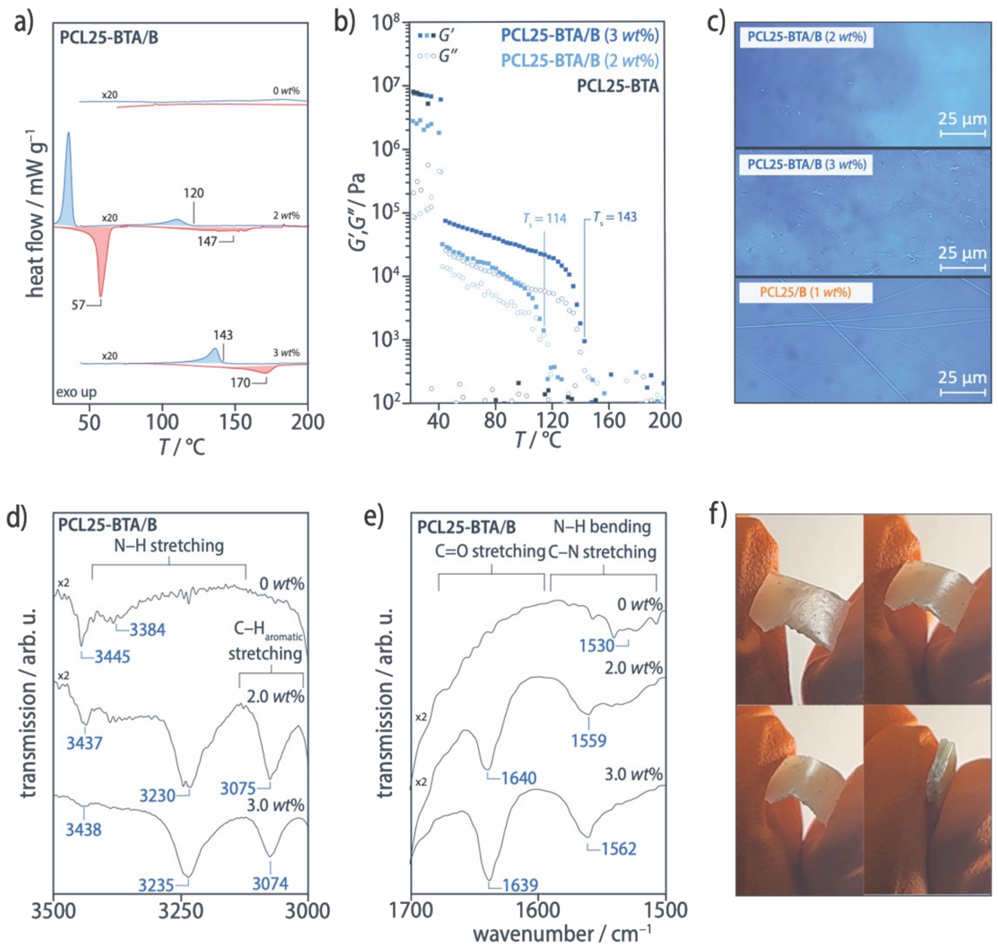

Supplementary Figure S17. *a)* Second DSC heating and cooling scans of **PCL25-BTA** recorded at 10 °C/min show no peaks other than the PCL crystallization and melting peaks (T_m = 57 °C), whereas scans of **PCL25-BTA/B** blends reveal additional transitions at $T_{d,NF}$ upon heating and T_{agg} upon cooling, corresponding to the dissociation and co-assembly of end-group-additive nanofibrils. *b)* The resulting nanofibrils are bridged by the polymer chains, resulting in a rubbery plateau in shear rheology cooling scans that extends from T_s, defined as the $G' > G''$ crossover temperature, down to T_c of the PCL matrix. *c)* **PCL25-BTA/B** with [**B**] < 3 *wt*% remains optically homogeneous in microscopy images recorded during cooling, until the PCL lamella start to form at T_c, whereas blends containing [**B**] ≥ 3 *wt*% show features characteristic of bulk **B** precipitates, although at much smaller volume fractions than in the corresponding PCL/**B** reference blends. In FTIR spectra of **PCL25-BTA/B**, *d)* the N–H stretching and *e)* C=O stretching band, and the combined N–H bending and C–N stretching band are characteristic of strongly hydrogen-bonded amides in extended BTA aggregates,[1] even at [**B**] < 3 *wt*%, where no features are visible in optical micrographs. By contrast, the bands in FTIR spectra of **PCL25-BTA** are characteristic of weakly aggregated BTA.[1] *f)* The low molar mass of the base polymer renders **PCL25-BTA/B** highly brittle.

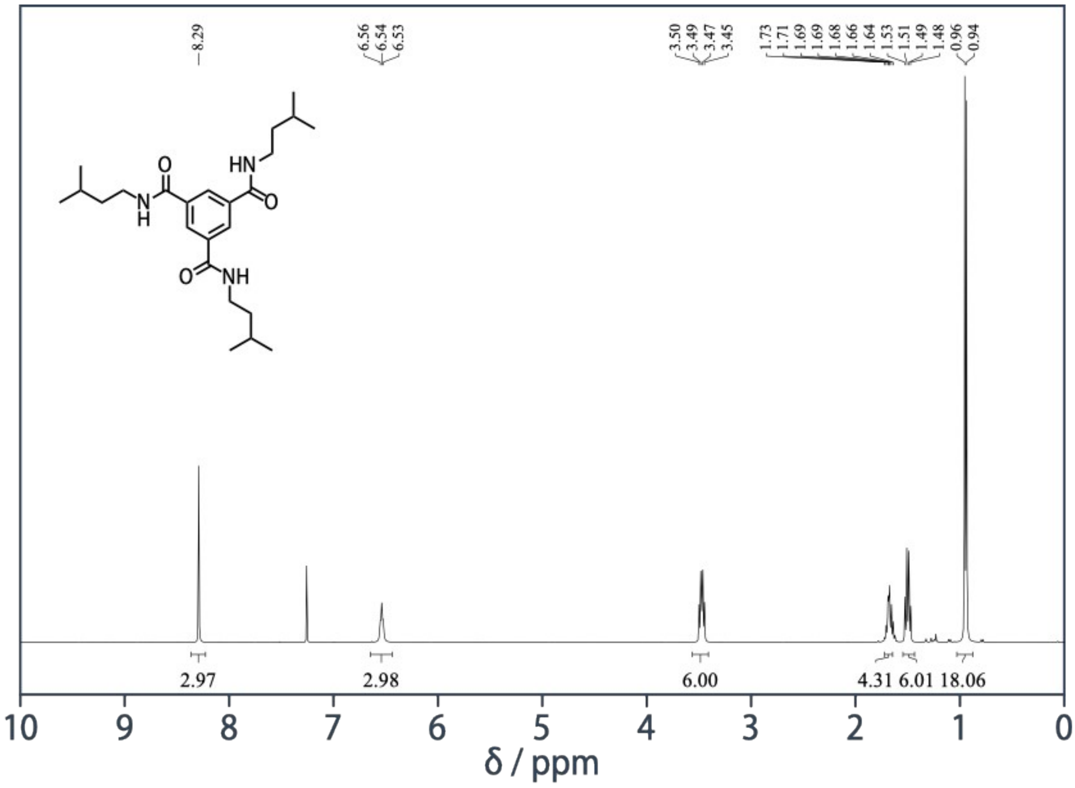

Supplementary Figure S18. ^{1}H NMR spectrum ($CDCl_3$, 400 MHz) of *N*,*N*,*N*-Tri(3-methylbutyl) benzene-1,3,5-tricarboxamide (**B**).

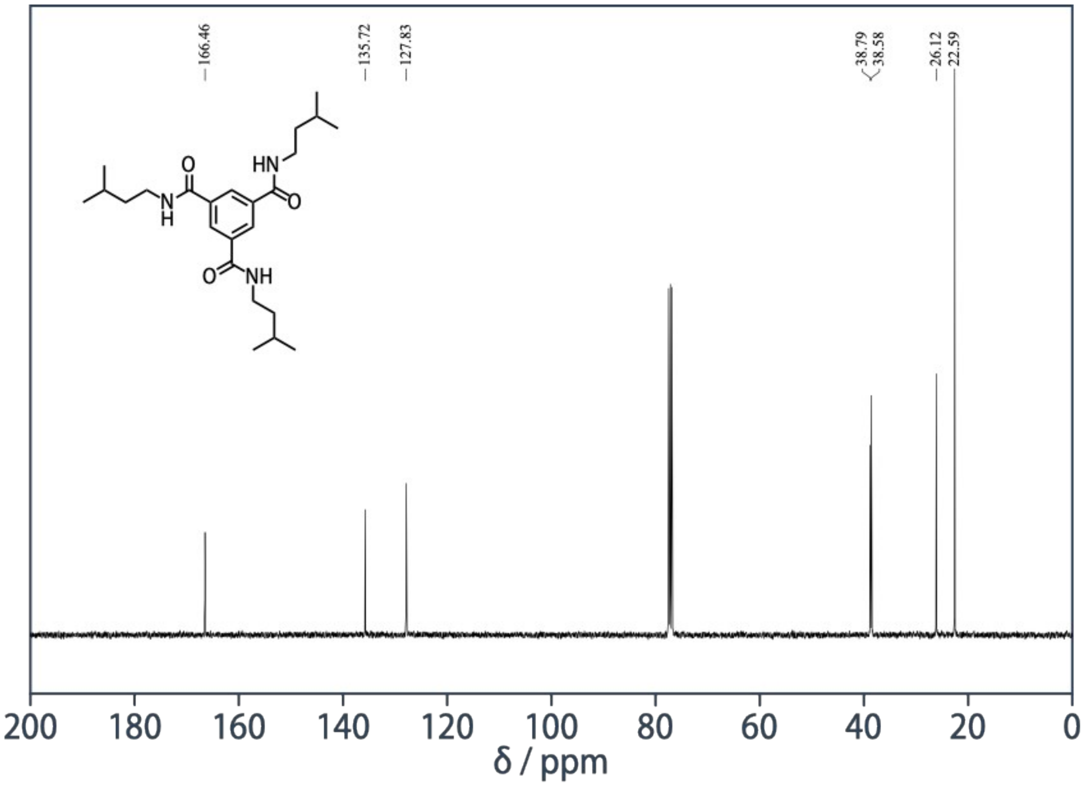

Supplementary Figure S19. ^{13}C NMR spectrum ($CDCl_3$, 100 MHz) of *N*,*N*,*N*-Tri(3-methylbutyl) benzene-1,3,5-tricarboxamide (**B**).

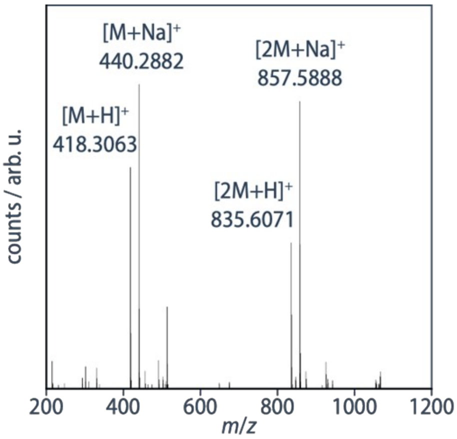

Supplementary Figure S20. HRMS spectrum of *N*,*N*,*N*-Tri(3-methylbutyl) benzene-1,3,5-tricarboxamide (**B**).

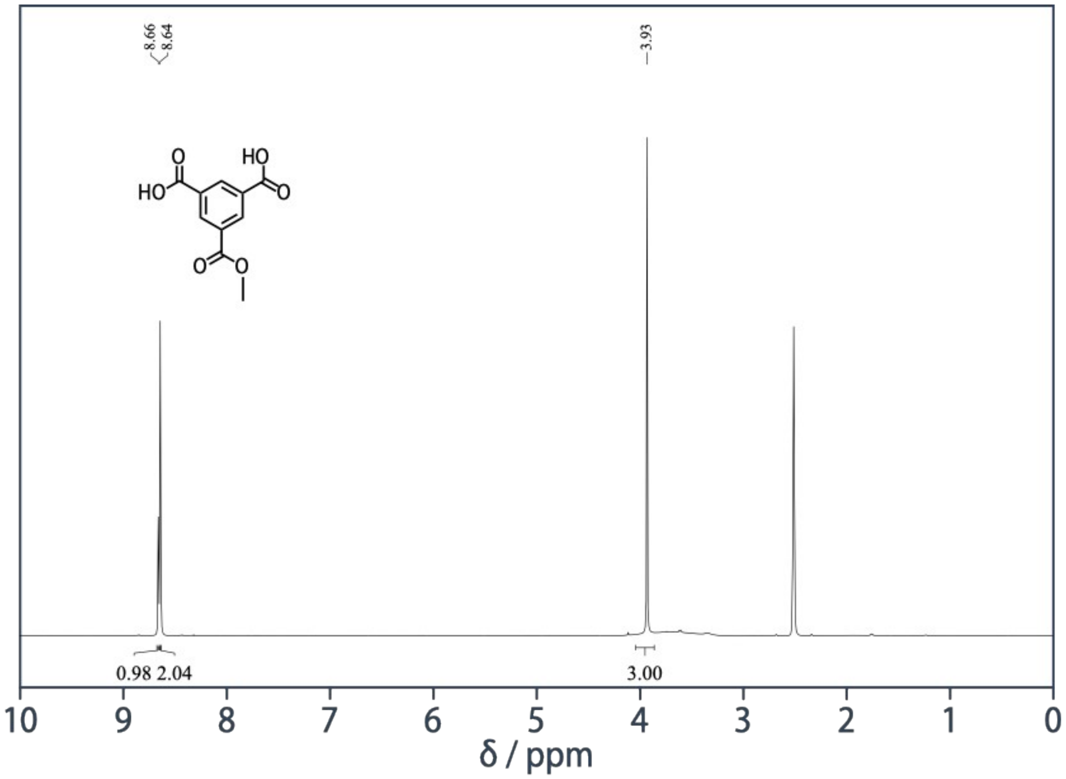

Supplementary Figure S21. ^{1}H NMR spectrum (DMSO-d_6, 400 MHz) of 5-methoxycarbonylbenzene-1,3-dicarboxylic acid (**1**).

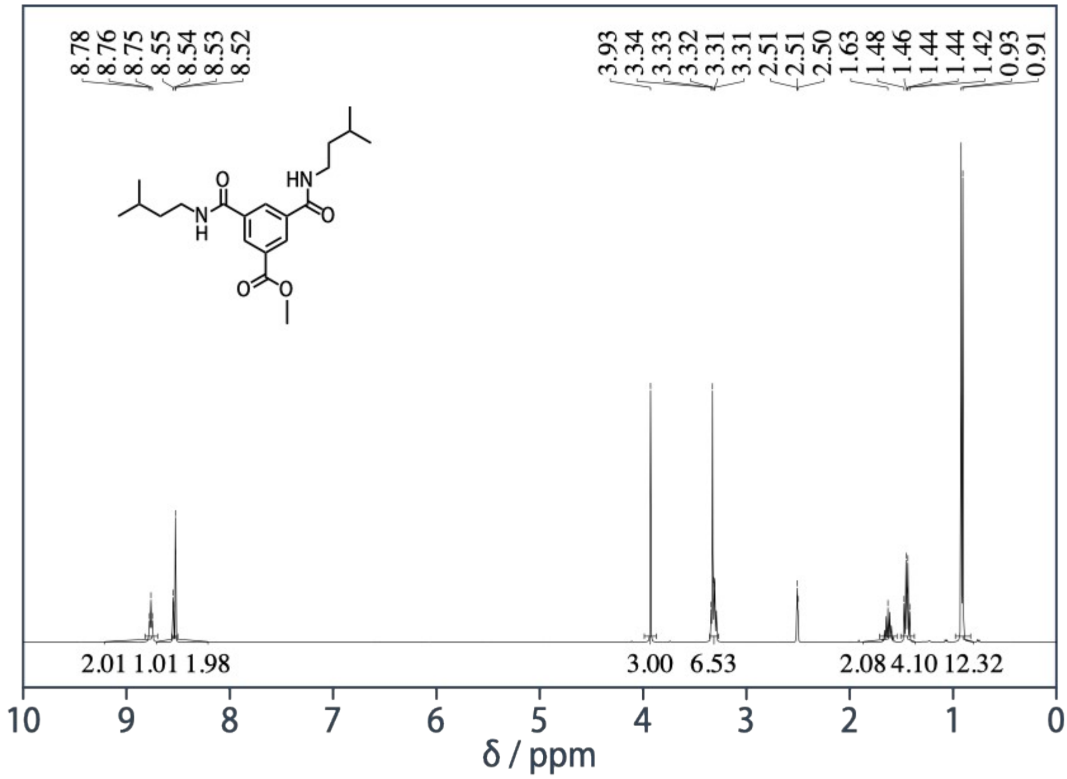

Supplementary Figure S22. ^{1}H NMR spectrum (DMSO-d_6, 400 MHz) of methyl 3,5-bis(3-methylbutylcarbamoyl)benzoate (**2**).

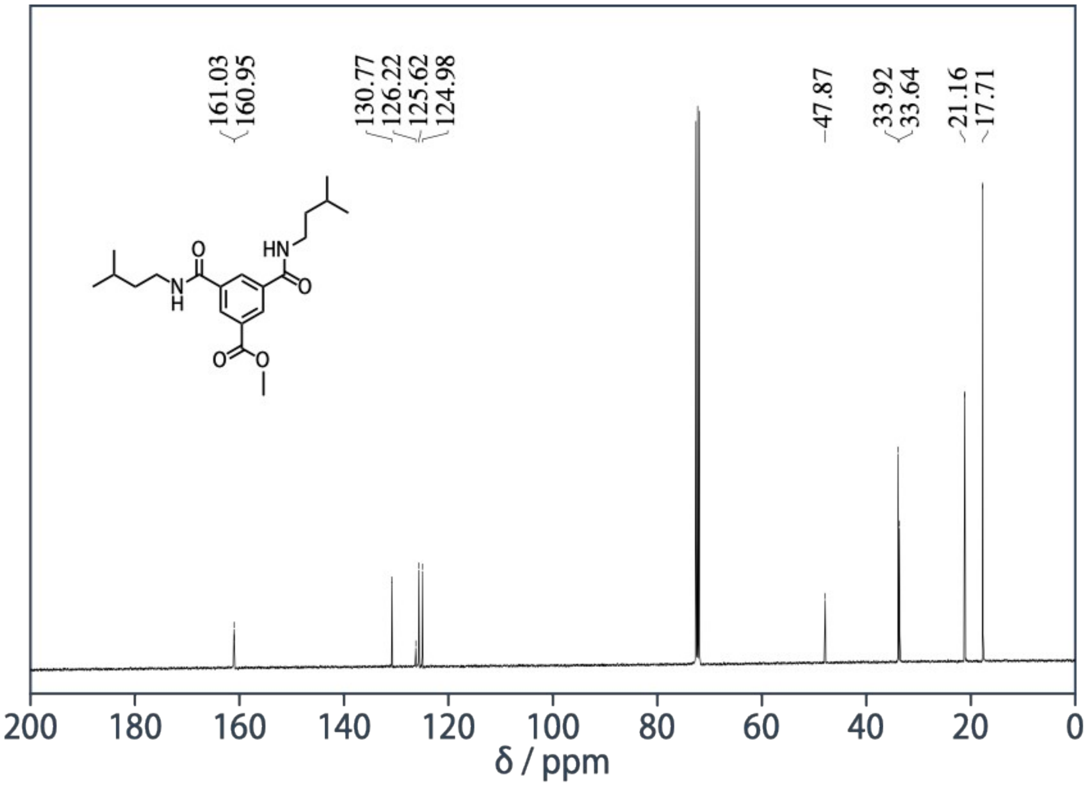

Supplementary Figure S23. ^{13}C NMR spectrum (DMSO-d_6, 100 MHz) of methyl 3,5-bis(3-methylbutylcarbamoyl)benzoate (**2**).

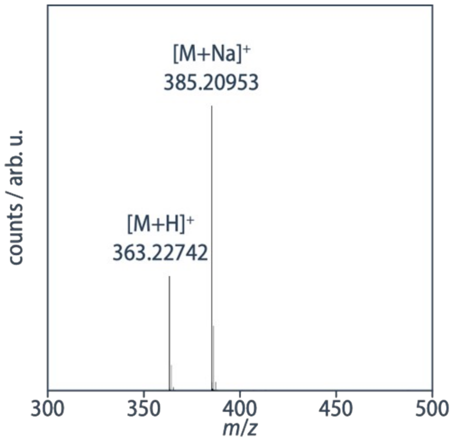

Supplementary Figure S24. HRMS spectrum of methyl 3,5-bis(3-methylbutylcarbamoyl)benzoate (**2**).

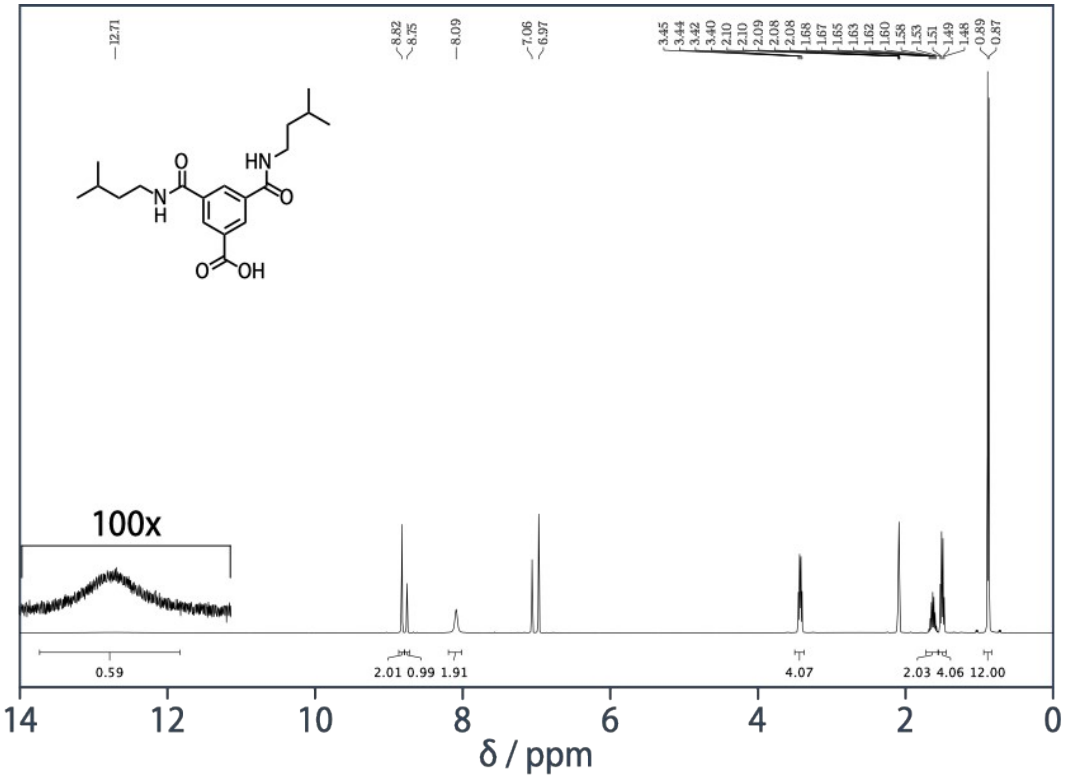

Supplementary Figure S25. ^{1}H NMR spectrum (Tol-d_8, 400 MHz, 375 K) of 3,5-bis(3-methylbutylcarbamoyl)benzoic acid (**3**).

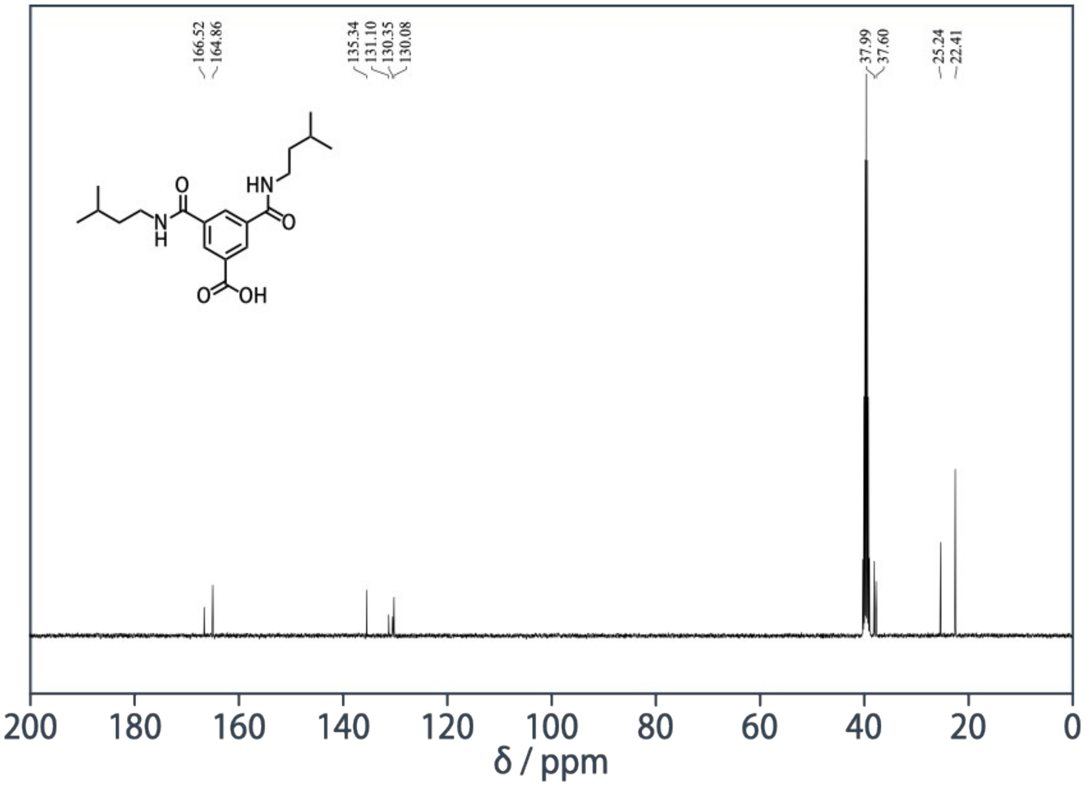

Supplementary Figure S26. ^{13}C NMR spectrum (DMSO-d_6, 100 MHz) of 3,5-bis(3-methylbutylcarbamoyl)benzoic acid (**3**).

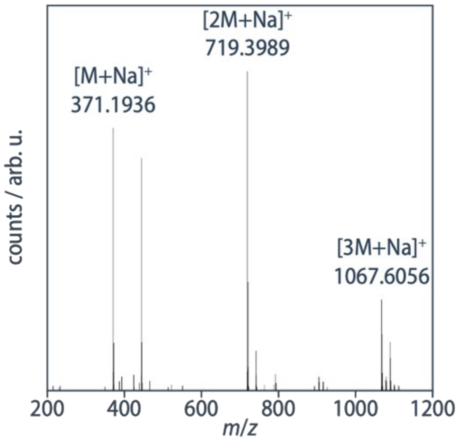

Supplementary Figure S27. HRMS spectrum of 3,5-bis(3-methylbutylcarbamoyl)benzoic acid (**3**).

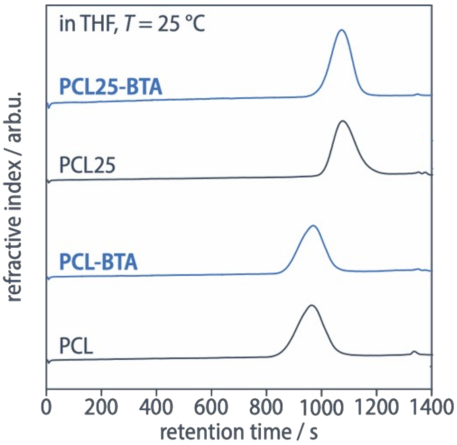

Supplementary Figure S28. GPC traces of **PCL25-BTA**, PCL25, **PCL-BTA** and PCL in THF. The molar mass remains the same after the end-modification of PCL.

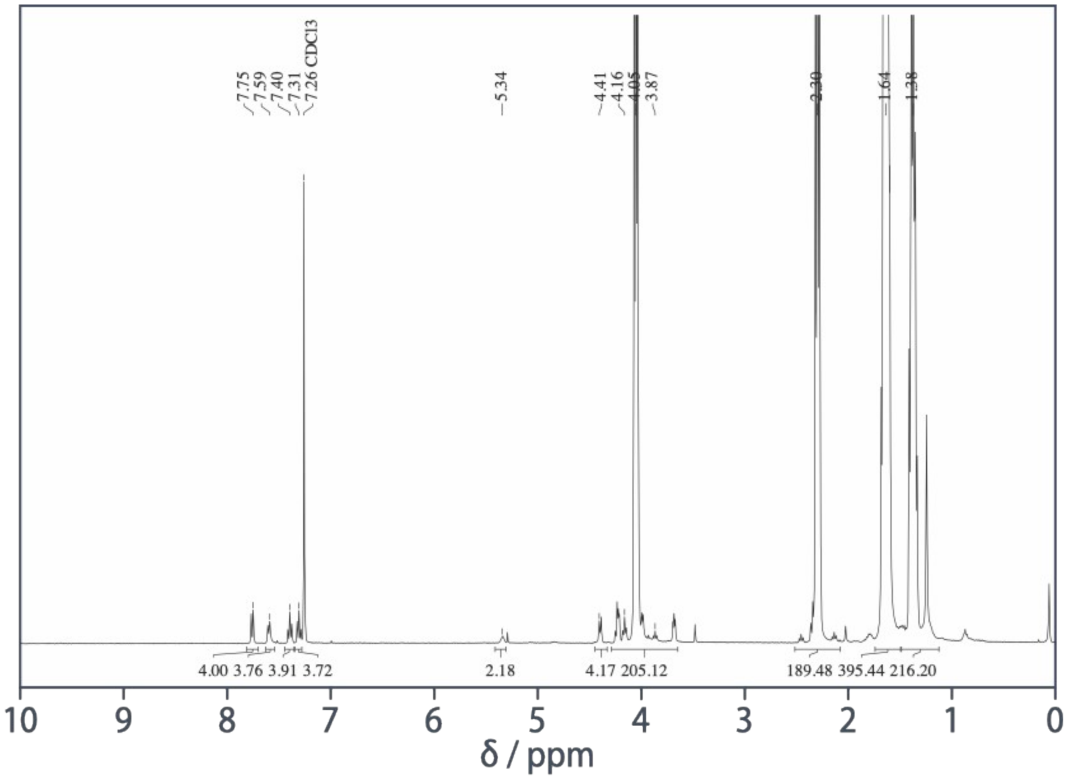

Supplementary Figure S29. ^{1}H NMR spectrum ($CDCl_3$, 400 MHz) of **PCL25-GlyFmoc**.

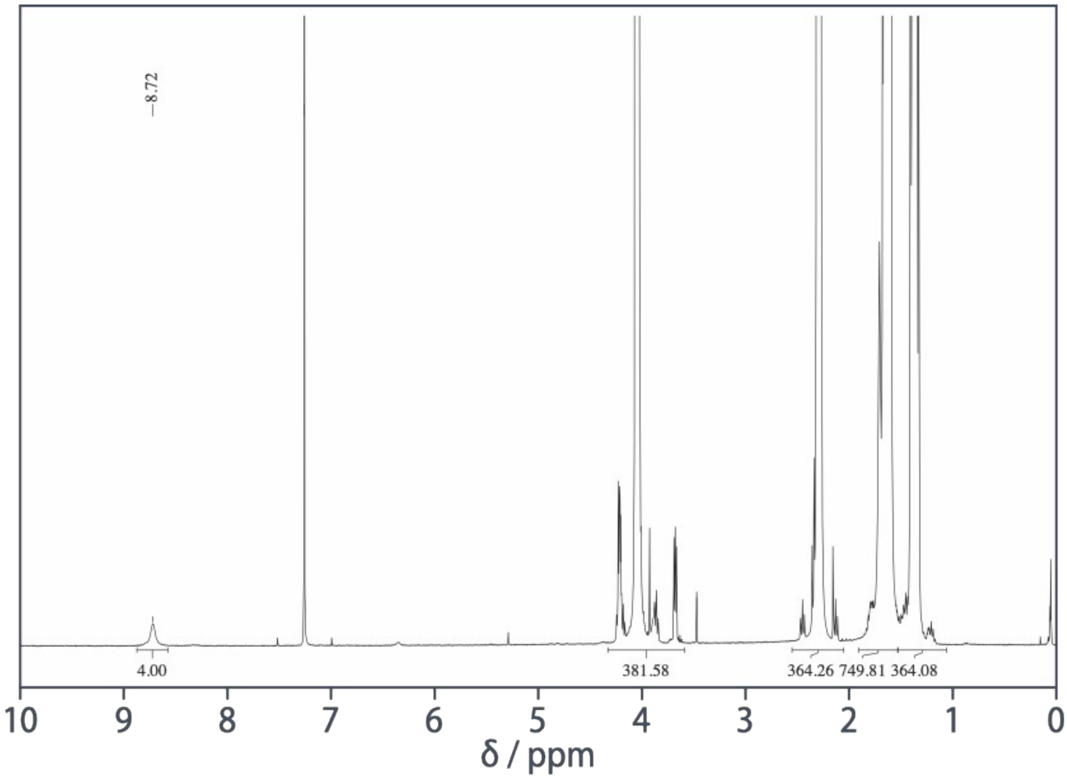

Supplementary Figure S30. ^{1}H NMR spectrum ($CDCl_3$, 400 MHz) of **PCL25-GlyNH$_2$**.

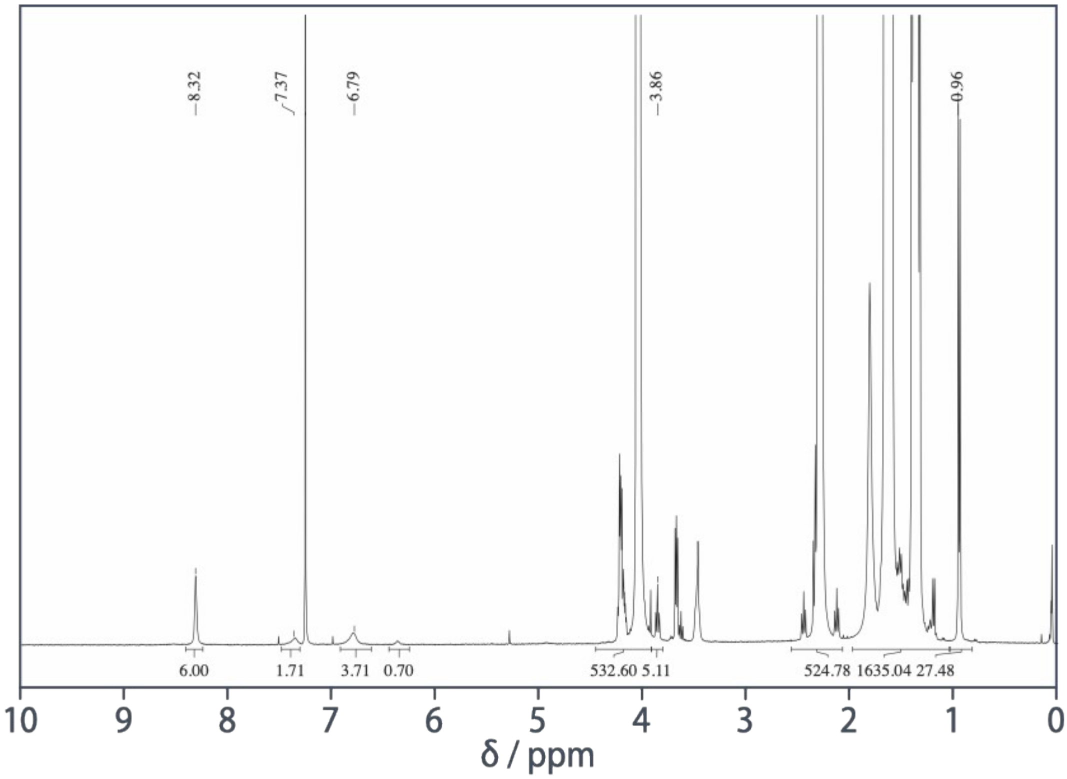

Supplementary Figure S31. ^{1}H NMR spectrum ($CDCl_3$, 400 MHz) of **PCL25-BTA**.

References

1 A. Timme, R. Kress, R. Q. Albuquerque and H.-W. Schmidt, Phase Behavior and Mesophase Structures of 1,3,5-Benzene- and 1,3,5-Cyclohexanetricarboxamides: Towards an Understanding of the Losing Order at the Transition into the Isotropic Phase, *Chem. – Eur. J.*, 2012, **18**, 8329–8339.

2 P. J. M. Stals, M. M. J. Smulders, R. Martín-Rapún, A. R. A. Palmans and E. W. Meijer, Asymmetrically Substituted Benzene-1,3,5-tricarboxamides: Self-Assembly and Odd–Even Effects in the Solid State and in Dilute Solution, *Chem. – Eur. J.*, 2009, **15**, 2071–2080.
